# Genome‐wide association study identifies QTL and candidate genes for grain size and weight in a *Triticum turgidum* collection

**DOI:** 10.1002/tpg2.20562

**Published:** 2025-01-27

**Authors:** G. Mangini, D. Nigro, P. L. Curci, R. Simeone, A. Blanco

**Affiliations:** ^1^ Institute of Biosciences and Bioresources, National Research Council Bari Italy; ^2^ Department of Soil, Plant and Food Sciences, Genetics and Plant Breeding Section University of Bari Aldo Moro Bari Italy

## Abstract

Wheat breeders are constantly looking for genes and alleles that increase grain yield. One key strategy is finding new genetic resources in the wild and domesticated gene pools of related species with genes affecting grain size. This study explored a natural population of *Triticum turgidum* (L.) phenotyped for grain weight and size‐related traits in three field trials and genotyped with single nucleotide polymorphism markers spread across the entire genome. The genome‐wide association study analysis identified 39 quantitative trait loci (QTL) for 1000‐kernel weight, grain length, grain width, grain area, and grain aspect consistent in at least two and across environments. Interestingly, 23 QTL for grain‐related traits were grouped in nine QTL clusters located on chromosomes 1A, 1B, 2B, 3B, 4B, 5A, and 6B, respectively. Moreover, most of these QTL support findings from previous QTL analyses and are further strengthened by the known functions of the genes (such as *BG2*, *GS5*, and *SRS3*) and their similarity to genes in other cereal species. QTL clusters harbored genes that participate in various metabolic processes potentially involved in seed development, phytohormone signaling, sugar transport, mitogen‐activated protein kinases signaling, and transcriptional factors (such as MADS‐box and WRKY). Identifying loci controlling grain‐related traits will provide information on the genetic resources available to breeders to improve grain yield, as well as the opportunity to develop close gene markers to be used in marker‐assisted selection programs.

## Core Ideas

- The tetraploid wheat germplasm showed a wide variation for grain weight and grain size‐related traits.
- The phylogenetic analysis splits the collection according to the van Slageren's *Triticum turgidum* classification.
- Twenty‐three out of 39 detected major quantitative trait loci (QTL) were identified in nine QTL clusters.
- QTL for grain size and weight include candidate genes involved in seed development and sugar transport.

**Abbreviations**
AREA: grain area
ASPECT: grain aspect
GL: grain length
GO: gene ontology
GW: grain width
GWAS: genome‐wide association study
LD: linkage disequilibrium
NJ: neighbor‐joining
PCA: principal component analysis
PVE: phenotypic variance explained
QTL: quantitative trait loci
SNP: single nucleotide polymorphism
TGW: thousand grain weight
TKW: 1000‐kernel weight

## 1 INTRODUCTION

Global wheat production was estimated at 808.4 million tons in 2022 (FAOSTAT, 2024), about 3.6% of which consisted of durum wheat (International Grains Council, 2023). Despite being a globally minor crop, durum wheat represents a major crop in the Mediterranean Basin, where its cultivation is widely spread and significantly contributes to cereal grain production. Other important durum wheat‐growing areas are the USA, Canada, and Mexico. Its grain is mainly used for pasta production in Southern Europe, couscous in North Africa, and typical products such as tabbouleh, freekeh, and bulgur in the Levant. Finely ground flour is also used for making pita bread and pizza.

Durum wheat belongs to *Triticum turgidum* (L.) Thell. (2*n* = 4*x* = 28, genome BBAA), which includes eight subspecies (van Slageren, 1994): one wild (ssp. *dicoccoides*, wild emmer) and seven domesticated ones. Among the latter, ssp. *dicoccum* (domesticated emmer) and ssp. *paleocolchicum* (Georgian wheat) are hulled forms; four are free‐threshing forms locally cultivated: ssp. *turgidum* (Rivet wheat, Cone, or Pollard wheat), ssp. *polonicum* (Polish wheat), ssp. *turanicum* (Khorasan wheat), and ssp. *carthlicum* (Persian wheat); the last one is represented by the major commercial free‐threshing form, ssp. *durum* (durum wheat). The wild emmer is morphologically similar and genetically closely related to the domesticated subspecies, and their F_1_ hybrids are fully or almost fully fertile. According to van Slageren's (1994) classification, all these tetraploid wheats are grouped into a single biological species (*T. turgidum*).

Archaeobotanical evidence proved that grain size was involved in the transition from wild to domesticated emmer, highlighting the strategic role of this trait in the wheat domestication syndrome (Fuller, 2007). Nowadays, grain size is a major selection and breeding target in modern wheat improvement programs, given its direct effects on grain yield. The pre‐anthesis phase affects grain size indirectly by modulating the source‐to‐sink relationship, where the “source” is the assimilation capacity determined by leaf area and “sink” represents the grain number per unit area. Indeed, the competition for assimilates between tillers and developing spike, any abiotic stress eventually occurring during microsporogenesis and pollen development, as well as the genetic factors involved in the spike and floret architecture, determine the final number of grains. These factors have an indirect influence on grain size and weight, which are generally negatively correlated with each other (Sadras, 2007). In post‐anthesis, grain cell division starts just after pollination, followed by growth through cell expansion (Jenner et al., 1991). Cell proliferation is positively correlated with grain weight and size (Long et al., 2023), confirming that these genes play a key role in the expression of grain traits (Calderini et al., 2021). The grain‐filling stage, depending on the assimilate supply derived both from current photosynthesis and remobilization of reserves from vegetative tissues, also affects grain size. The grain‐filling duration mostly depends on environmental conditions (Teng et al., 2023), whereas the grain‐filling rate is strongly determined by the genotype (Baillot et al., 2018).

Grain size can be specified by grain length (GL), grain width (GW), grain aspect (ASPECT), grain area (AREA), and grain weight expressed as a 1000‐kernel weight (TKW). GL and GW are determined by cell number and cell size, while ASPECT, area, and TKW are mostly affected by the grain‐filling process (Bai et al., 2012). Grain size‐related traits and grain weight are complex quantitative traits controlled by several quantitative trait loci (QTL)/genes influenced by environmental factors and with a significant genotype × environment interaction. Linkage mapping and genome‐wide association study (GWAS) identified and mapped over 100 QTL for grain weight and size distributed across nearly all wheat chromosomes (reviews by Cao et al., 2020; Saini et al., 2022; Taranto, Esposito, & De Vita, 2023). Recent molecular investigations have identified and cloned some major candidate genes for thousand grain weight (TGW), GW, and GL. Gasparis and Miłoszewski (2023) presented a comprehensive analysis of the genetic and molecular factors influencing grain size and weight in wheat, rice, and barley; the same authors provided a list of genes associated to their molecular mechanisms and regulatory pathways affecting grain size‐related traits. In durum wheat, several QTL have been identified by linkage mapping using different biparental mapping populations (Desiderio et al., 2019; Mangini et al., 2021; Russo et al., 2014; Valladares García et al., 2023). However, direct comparisons of QTL identified in different studies are difficult due to the limited number of common markers between mapping populations. In addition, QTL mapping is highly dependent on the genetic diversity of the two parents, and the effects of the detected QTL can vary between populations. Furthermore, QTL regions can be quite large, comprising too many loci to investigate for the identification of candidate genes. The limitations of linkage analysis can be overcome by GWAS using natural germplasm, which can narrow down the candidate regions. GWAS is based on linkage disequilibrium (LD) and provides a much higher resolution capacity to capture insights into the genetic architecture of complex traits than traditional linkage QTL mapping (Scherer & Christensen, 2016). GWAS approaches were mainly used in durum collections to identify genomic regions or candidate genes involved in grain size (Alemu et al., 2020; Groli et al., 2024; Jia et al., 2024; Mulugeta et al., 2023; Sesiz, 2023; Sun et al., 2020; Taranto, Esposito, Fania et al., 2023). Therefore, extending GWAS to tetraploid wheat collections, including wild, domesticated, and cultivated forms, could represent a strategic tool to detect QTL and candidate genes or unexplored alleles controlling grain size and weight.

In recent years, with the development of high‐throughput genotyping technology, single nucleotide polymorphism (SNP) arrays have become a powerful tool for GWAS in durum wheat (Laribi et al., 2023; Mangini et al., 2018; Nigro et al., 2019). Moreover, the availability of high‐quality reference genome sequences for wild emmer and durum wheat (Avni et al., 2017; Maccaferri et al., 2019) provides powerful support for deciphering marker‐trait associations (MTA) and simplifying the search and discovery of candidate genes underlying grain size and grain weight. In this study, a panel of 165 tetraploid wheat accessions was genotyped by the 90K iSelect array and evaluated for grain size and weight in three field trials. GWAS analysis was performed for five grain‐related traits to identify QTL and candidate genes. Identifying loci controlling grain‐related traits will provide information on the genetic resources available to breeders to improve grain yield, as well as the opportunity to develop closely associated markers to be used in marker‐assisted selection (MAS) programs.

**Core Ideas**

- The tetraploid wheat germplasm showed a wide variation for grain weight and grain size‐related traits.
- The phylogenetic analysis splits the collection according to the van Slageren's *Triticum turgidum* classification.
- Twenty‐three out of 39 detected major quantitative trait loci (QTL) were identified in nine QTL clusters.
- QTL for grain size and weight include candidate genes involved in seed development and sugar transport.

## 2 MATERIALS AND METHODS

### 2.1 Germplasm and grain size phenotyping

A total of 165 accessions of *Triticum turgidum* L. (2*n* = 4*x* = 28; BBAA genome) were grown in field trials at Valenzano (Bari, Italy) for 3 years (2010, 2013, and 2014). The collection included seven *T. turgidum* subspecies: ssp. *durum* (72 accessions), ssp. *turanicum* (21), ssp. *polonicum* (15), ssp. *turgidum* (17), ssp. *carthlicum* (12), ssp. *dicoccum* (17), and ssp. *dicoccoides* (11) (Table S1). A randomized complete block design with two replications and plots consisting of a single row of 1 m length, 60 cm apart, with 85 seeds per plot, was used. The field experiments were supplied with 45 kg/ha N and 115 kg/ha P_2_O_5_ in pre‐sowing and 85 kg/ha N in top dressing. At maturity, all the spikes of each plot were hand‐harvested and bulk‐threshed by a microthresher. Grain‐related traits were determined on 10 g of grains for each replication of each accession by using high‐resolution scanner‐based image analysis. The images were processed using the Image‐Pro Plus 7.0 software (Media Cybernetics). GL, GW, ASPECT (ratio between the major axis and the minor axis of the ellipse equivalent to the grain), AREA, and grain number were measured. The grain number was used to calculate the TKW.

Analysis of variance (ANOVA) was performed to test the significance of differences among accessions and replications. Combined analysis across the three trials was carried out for all grain‐related traits. ANOVA, principal component analysis (PCA), descriptive statistical analysis, and Pearson correlation coefficients analysis among different traits were calculated in R (http://www.r‐project.org). Broad‐sense heritability (*h* ^2^_B_) for each trait and the best linear unbiased estimator (BLUE) values were calculated using IciMapping v. 4.2 (Meng et al., 2015).

### 2.2 SNP genotyping

Genomic DNA was isolated from freeze‐dried young leaf tissues using the protocol described by Sharp et al. (1988). After quality and quantity check, the DNA was diluted to 50 ng/µL and genotyped with the wheat 90K iSelect array (S. Wang et al., 2014) by TraitGenetics GmbH (https://sgs‐institut‐fresenius.de/en/health‐nutrition/traitgenetics) following the manufacturer's recommendations as described in Akhunov et al. (2009). The genotyping assays were carried out using the Illumina iScan reader and performed using GenomeStudio software version 2011.1 (Illumina). The durum wheat consensus map developed by Maccaferri et al. (2015) was used to order markers.

### 2.3 Phylogenetic analysis

The genotypic data were filtered, discarding SNPs with >10% missing data points and markers with a minimum allele frequency of <5%. A total of 15,211 polymorphic markers were retained and used for subsequent analyses. A phylogenetic tree was constructed based on the distance matrix estimated by the SNP dataset with the neighbor‐joining (NJ) clustering method in TASSEL software version 5.0 (Bradbury et al., 2007). The resulting tree was visualized using FigTree v.1.4.4 (Rambaut, 2018).

### 2.4 Association mapping and linkage disequilibrium estimation

The filtered genotypic data and the phenotypic mean values of each field trial, as well as the overall BLUE mean across the three trials, were used for the GWAS analysis. GWAS was performed using the mixed linear model (MLM) including the population structure (PCA) and the kinship (K) similarity matrix (MLM + PCA + K) in TASSEL software version 5.0 (Bradbury et al., 2007). A threshold *p*‐value of 0.001 (−log10[*p*] ≥ 3.0) was used to declare significant MTA. Suggestive MTA at the sub‐threshold 2.5 ≤ −log10(*p*) ≤ 2.9 are reported only for QTL declared significant at −log10(*p*) ≥ 3.0 in at least two environments and across environments. The phenotypic variation (*R* ^2^) and additive effect were estimated for each MTA. The positive or negative additive value refers to the MTA allele with higher frequency. The International Rules of Genetic Nomenclature for wheat were used for QTL designation, and the software MapChart v. 2.2 was used for the graphical representation of linkage groups and QTL.

LD was calculated using the SNP markers mapped in the durum wheat Svevo reference genome. The physical SNP positions were determined according to Maccaferri et al. (2019) or by Basic Local Alignment Search Tool (BLAST)‐ing the 100 bp sequences, including the SNP, against the durum genome. LD was estimated as allele frequency correlation (*r* ^2^) between pairwise SNPs using the TASSEL software version 5.0 (Bradbury et al., 2007). Using TASSEL output, the *r* ^2^ values were plotted against the genetic distance, and the locally weighted polynomial regression (LOESS) curve was drawn to determine, according to Remington et al. (2001), the LD decay by a custom R script in R software. LD decay was identified as the physical genomic distance at which the *r* ^2^ decreased to half of its maximum value, where *r* ^2^ = 1, indicating complete LD, and *r* ^2^ = 0, indicating absence of LD.

### 2.5 SNP annotation and gene ontology analysis

Gene annotation within confidence intervals (CIs) of each peak marker was performed using the Svevo durum wheat high‐confidence gene models (https://www.interomics.eu/durum‐wheat‐genome). The CI size within SNP flanking regions was determined based on LD decay. In order to detect potential candidate genes within each region, functional annotations were screened according to literature data reporting candidate genes involved in grain size control in both rice and bread wheat, and durum wheat orthologous genes were searched within each QTL. In case durum wheat orthologous did not map within the target QTL intervals, their paralogues genes were additionally searched, and their physical localization within QTL identified in this paper was checked. Furthermore, to identify gene ontology (GO) terms associated with positional candidate genes detected in the tetraploid wheat collection and to find the over‐representation of a given GO term in a subset in comparison with the genome‐wide background frequency, enrichment analyses were performed using g:Profiler (Kolberg et al., 2023) with default parameters. In order to obtain the finest and most precise analysis due to the higher annotation quality in bread wheat, the GO analysis was performed by launching in the tool the corresponding bread wheat orthologous genes for each durum wheat candidate retrieved in the CI as previously described. Bread wheat orthologous genes were retrieved using BioMart from Ensembl Plants (https://plants.ensembl.org) by querying all the durum wheat genes identified as described above. Specifically, the GO analysis was carried out by grain traits (TKW, AREA, GL, GW, and ASPECT), querying all at once the orthologous bread wheat candidate genes for each QTL identified for a specific trait.

The g:SCS method (Reimand et al., 2007) was used as the default method for computing multiple testing corrections for *p*‐values gained from GO and pathway enrichment analysis, corresponding to an experiment‐wide threshold of *α* = 0.05.

## 3 RESULTS

### 3.1 Phenotypic variation for grain traits

**FIGURE 1:**
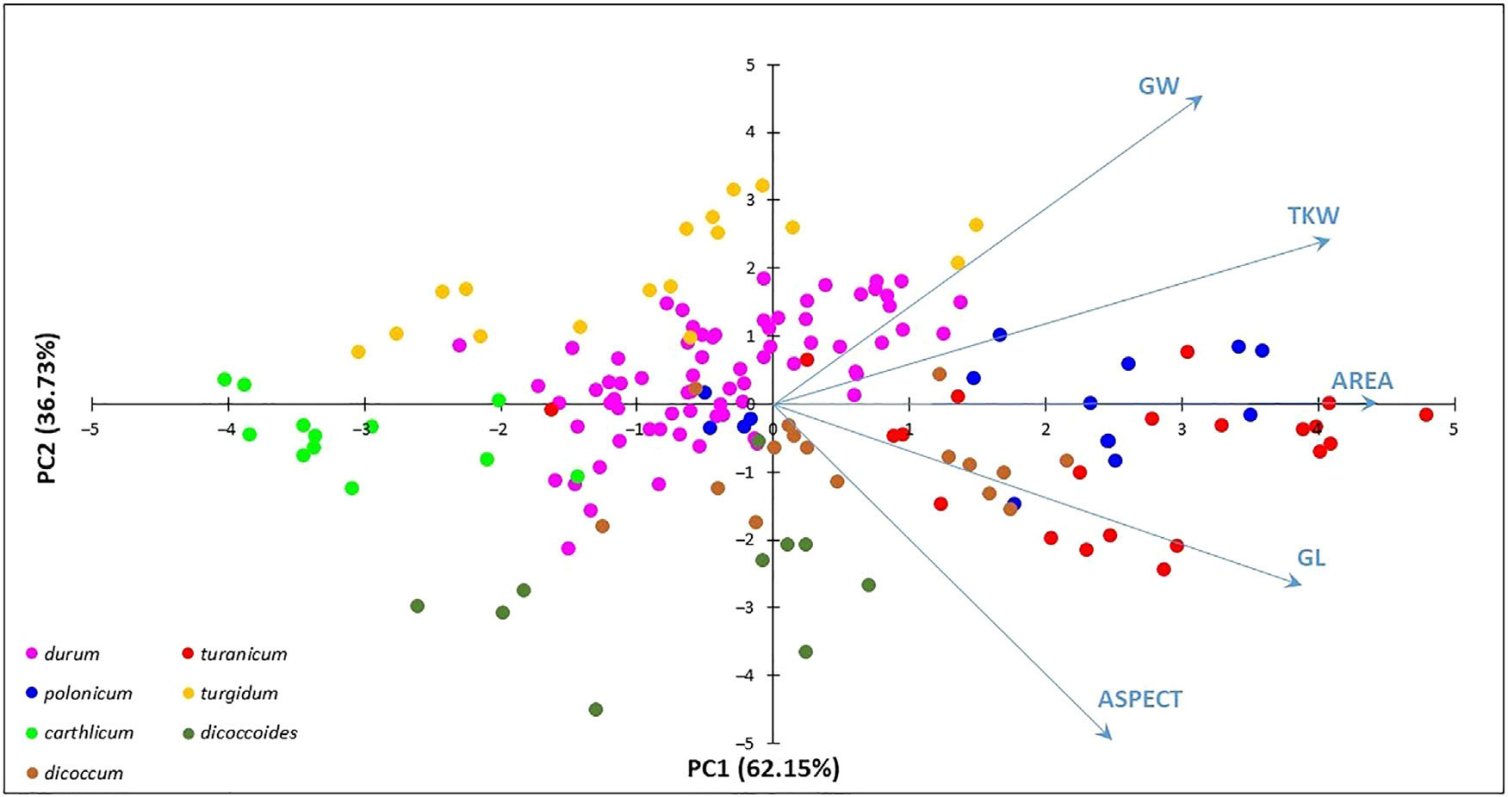
Scatter plot of the first two principal components from analysis of 1000‐kernel weight (TKW), area (AREA), grain length (GL), grain width (GW), and grain aspect (ASPECT) in a tetraploid wheat collection. The wheat accessions are shown as points colored according to van Slageren's taxonomic classification of *Triticum turgidum* subspecies. The percentages in each axis represent the proportion of variance explained by each principal component (PC).

A tetraploid wheat collection was evaluated for TKW, AREA, GL, GW, and ASPECT in replicated field trials carried out at Valenzano (Italy) over 3 years (2010, 2013, and 2014). ANOVA revealed highly significant differences (*p* ≤ 0.001) between genotypes for all grain‐related traits in each environment (Table S2). The combined analysis across environments revealed significant effects of genotypes, environments, and a strong genotype × environment interaction (Table S3). PCA based on TKW and grain size‐related traits mean values splits the collection according to van Slageren's (1994) *T. turgidum* subspecies classification (Figure 1). The first component discriminated the accessions of the ssp. *carthlicum*, ssp. *durum*, ssp. *polonicum*, and ssp. *turanicum*; and the second one distinguished the ssp. *turgidum*, ssp. *durum*, ssp. *dicoccum*, and ssp. *dicoccoides* accessions. The first two components explained over 95% of phenotypic variation.

**FIGURE 2:**
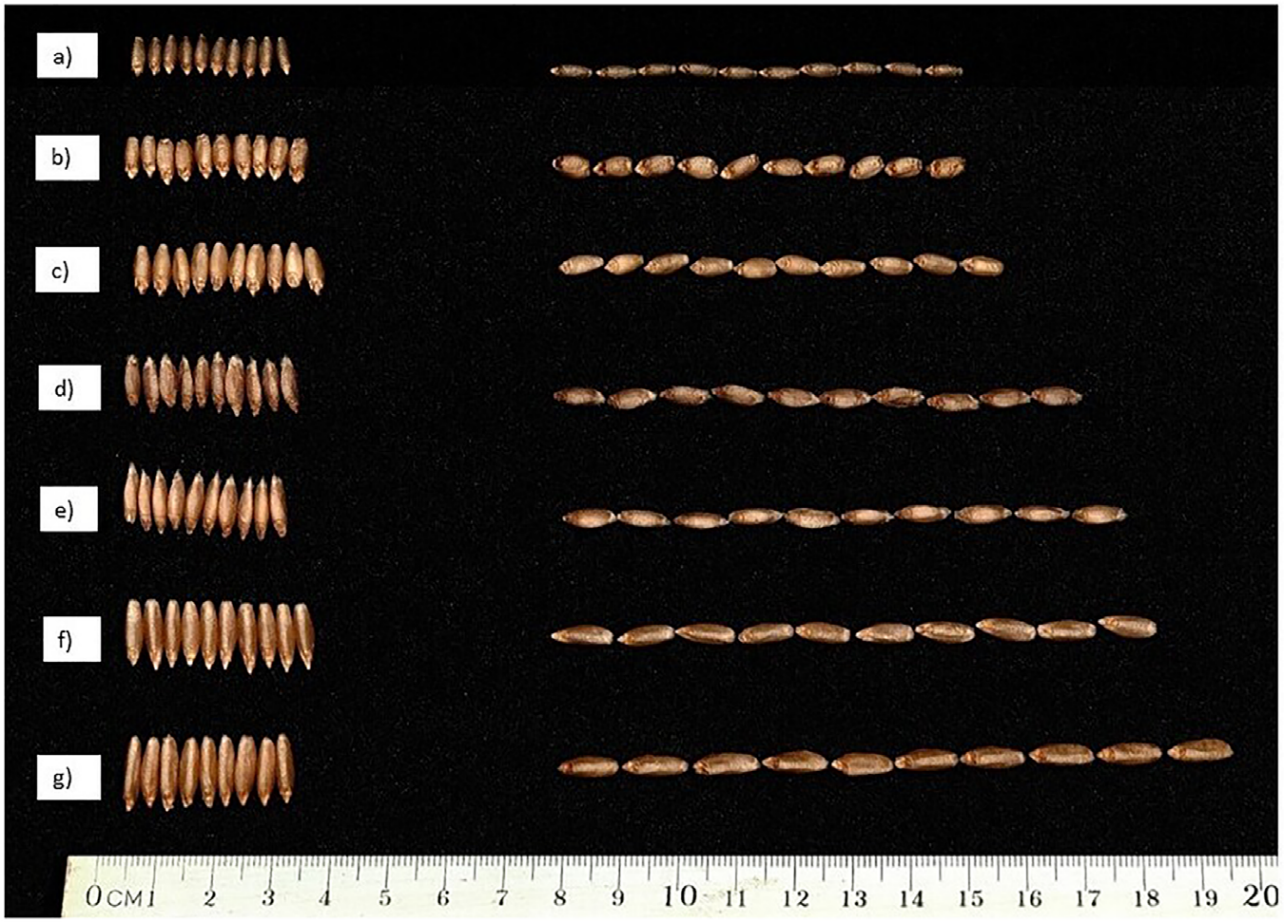
Grain size of representative accessions belonging to seven *Triticum turgidum* subspecies. (a) ssp. *carthlicum* (PI573182), (b) ssp. *turgidum* (PI157985), (c) ssp. *durum* (cultivar Iride), (d) ssp. *dicoccoides* (MG5444), (e) ssp. *dicoccum* (MG5350), (f) ssp. *polonicum* (PI330554), (g) ssp. *turanicum* (PI192641).

The mean values across years, range of variation, coefficient of variation, and heritability (*h* ^2^_B_) of TKW and grain size‐related traits are reported in Table 1. Wheat accessions are grouped by subspecies and the cultivated durum are split into “landraces or old cultivars” (released before 1973) and “modern cultivars” (released after 1973), respectively. In the whole collection, a large variation was observed for TKW (26.1–81.3 g), AREA (12.8–29.6 mm^2^), and GL (12.8–29.6 mm) with average values of 48.9 g, 20.6 mm, and 8.1 mm, respectively (Figure 2). The ssp. *turanicum* accessions had the highest mean value for TKW (62.0 g), AREA (25.4 mm^2^), and GL (9.7 mm), while the ssp. *carthlicum* showed the lowest average values (31.6 g, 14.6 mm^2^, and 6.5 mm, respectively). GW had a range of variation from 2.5 to 3.8 mm in the whole collection. Among the different subspecies, ssp. *dicoccoides* and ssp. *carthlicum* had the lowest GW mean (2.8 and 2.9 mm, respectively), while the other subspecies showed similar GW mean values ranging from 3.2 mm (ssp. *dicoccum*) to 3.5 mm (ssp. *turgidum*). The ASPECT range of variation was 1.7–3.7 in the whole collection, the ssp. *dicoccoides* accessions showing the highest mean value (3.2). The durum cultivars and the ssp. *carthlicum* accessions showed the lowest mean value (2.3).

**TABLE 1:** Mean values, standard error, range of variation (minimum and maximum), coefficient of variation (CV) and heritability (*h* ^2^_B_) of 1000‐kernel weight (TKW), area (AREA), grain length (GL), grain width (GW), and grain aspect (ASPECT) in a tetraploid wheat collection evaluated at Valenzano (Bari, Italy) for 3 years (2010, 2013, and 2014).

Trait	*Triticum turgidum* ssp.^a^	Mean	Standard error	Minimum	Maximum	CV (%)	*h* ^2^_B_
TKW (g)	Whole collection (165)	48.9	1.09	26.1	81.3	23.1	0.94
	ssp. *durum* (72)	48.5	0.75	37.0	63.5	13.0	
	Landraces and old cultivars (26)	46.2	1.48	37.0	63.5	16.4	
	Modern cultivars (46)	49.8	0.76	40.0	61.3	10.3	
	ssp. *turanicum* (21)	62.0	2.46	37.0	81.3	17.9	
	ssp*. polonicum* (15)	59.6	2.58	44.5	74.0	16.8	
	ssp. *turgidum* (17)	49.4	2.40	34.4	67.5	20.1	
	ssp. *carthlicum* (12)	31.6	1.09	28.3	38.7	11.9	
	ssp. *dicoccum* (17)	46.8	1.67	35.4	60.9	14.8	
	ssp. *dicoccoides* (11)	33.9	2.01	26.1	42.2	19.7	
AREA (mm^2^)	Whole collection	20.6	0.32	12.8	29.6	16.9	0.97
	ssp. *durum*	19.8	0.19	16.2	23.0	8.1	
	Landraces and old cultivars	19.1	0.38	16.2	23.0	10.1	
	Modern cultivars	20.1	0.19	17.8	22.3	6.3	
	ssp. *turanicum*	25.4	0.64	17.7	29.6	11.4	
	ssp*. polonicum*	24.1	0.73	19.7	27.8	11.7	
	ssp. *turgidum*	19.6	0.47	0.2	29.6	30.1	
	ssp. *carthlicum*	14.6	0.44	12.8	17.7	10.5	
	ssp. *dicoccum*	22.0	0.49	18.4	25.0	9.1	
	ssp. *dicoccoides*	19.3	0.71	15.2	22.4	12.2	
GL (mm)	Whole collection	8.1	0.11	5.6	10.7	14.1	0.98
	ssp. *durum*	7.7	0.04	6.4	8.3	4.6	
	Landraces and old cultivars	7.6	0.08	6.4	8.3	5.6	
	Modern cultivars	7.7	0.04	7.1	8.3	3.9	
	ssp. *turanicum*	9.7	0.21	7.3	10.7	9.6	
	ssp*. polonicum*	9.1	0.20	7.7	10.1	8.5	
	ssp. *turgidum*	7.5	0.18	0.0	10.7	29.9	
	ssp. *carthlicum*	6.5	0.17	5.6	7.7	9.0	
	ssp. *dicoccum*	8.8	0.10	7.9	9.5	4.5	
	ssp. *dicoccoides*	8.8	0.20	7.6	9.6	7.6	
GW (mm)	Whole collection	3.3	0.04	2.5	3.8	8.3	0.94
	ssp. *durum*	3.3	0.02	2.7	3.6	6.2	
	Landraces and old cultivars	3.2	0.05	2.7	3.6	8.2	
	Modern cultivars	3.3	0.02	3.1	3.6	4.6	
	ssp. *turanicum*	3.3	0.04	3.1	3.6	5.0	
	ssp*. polonicum*	3.4	0.04	3.2	3.7	5.1	
	ssp. *turgidum*	3.2	0.08	0.0	8.2	31.1	
	ssp. *carthlicum*	2.9	0.03	2.7	3.1	3.2	
	ssp. *dicoccum*	3.2	0.04	2.9	3.5	5.6	
	ssp. *dicoccoides*	2.8	0.07	2.5	3.2	8.4	
ASPECT	Whole collection	2.5	0.04	1.7	3.7	15.9	0.97
	ssp. *durum*	2.3	0.02	2.0	2.9	7.9	
	Landraces and old cultivars	2.4	0.05	2.0	2.9	10.6	
	Modern cultivars	2.3	0.02	2.1	2.6	5.7	
	ssp. *turanicum*	2.9	0.06	2.3	3.4	10.0	
	ssp*. polonicum*	2.7	0.05	2.3	3.0	7.9	
	ssp. *turgidum*	2.5	0.11	0.0	10.6	53.4	
	ssp. *carthlicum*	2.3	0.06	2.0	2.6	8.5	
	ssp. *dicoccum*	2.8	0.03	2.6	3.0	3.8	
	ssp. *dicoccoides*	3.2	0.09	2.6	3.7	9.5	

^a^ Number of accessions of whole collection and of *Triticum turgidum* ssp. reported in round brackets of TKW.

**FIGURE 3:**
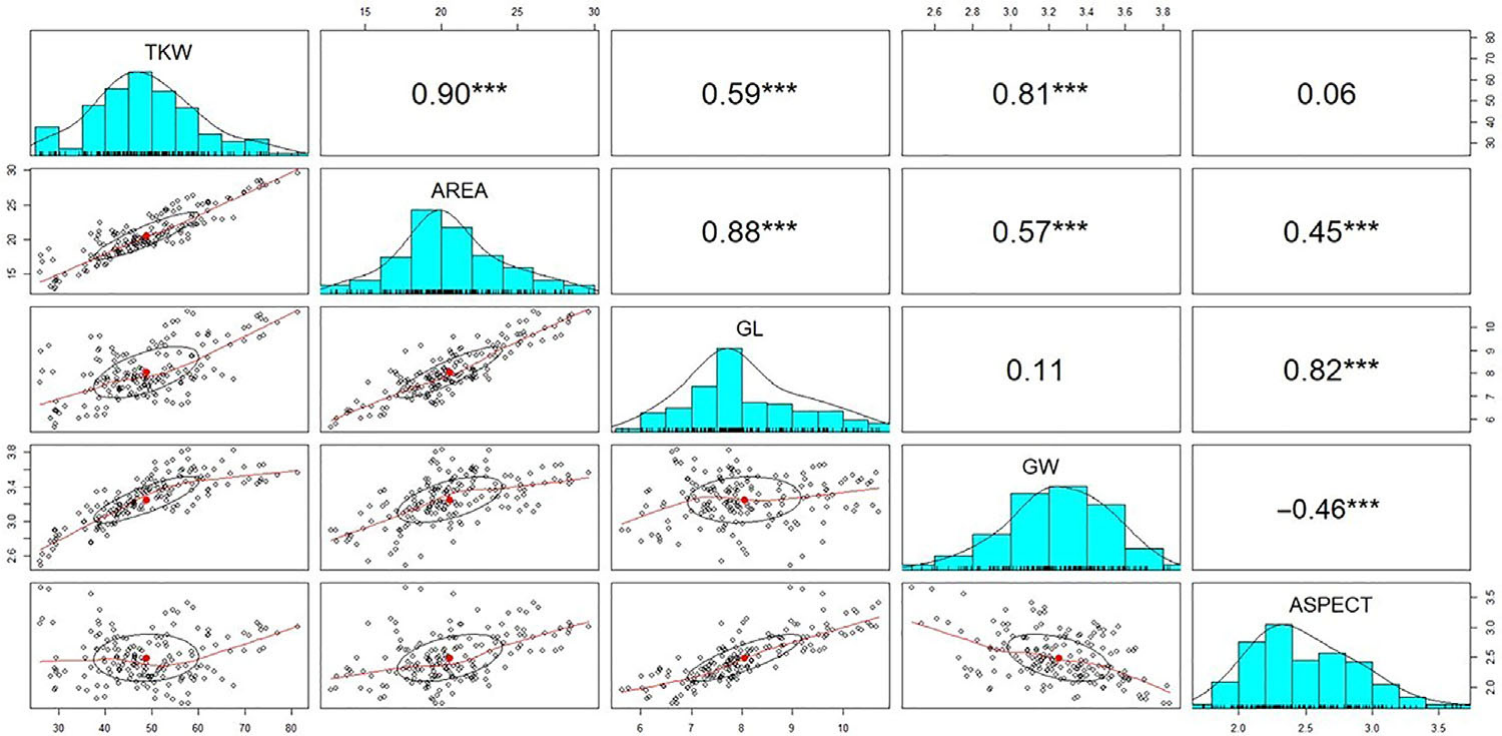
Pairwise scatter plot, histogram, and Pearson correlation analysis on 1000‐kernel weight (TKW), area (AREA), grain length (GL), grain width (GW), and grain aspect (ASPECT) in a tetraploid wheat collection evaluated at Valenzano (Bari, Italy) for 3 years (2010, 2013, and 2014). The diagonal panel shows the histograms for each grain trait. The lower and upper triangular panels, respectively, show scatter plots and Pearson correlation coefficients between the grain‐related traits. ***Significant at the *p* ≤ 0.001 level.

High values of broad‐sense heritability (*h* ^2^_B _> 0.90) were found for all grain‐related traits in individual environments and across environments (Tables S2 and S3). The frequency distribution pattern (Figure 3) and correlations of grain‐related traits between environments (Table S4) confirmed the large variation observed among the *T*. *turgidum* subspecies. In addition, as expected, phenotypic correlation analysis revealed highly significant (*p* ≤ 0.001) and positive relationships among TKW, AREA, GL, and GW across years (Figure 3) and in each year (Table S5). GW resulted negatively related (*p* ≤ 0.001) to ASPECT in the three field experiments (*r* values ranging from −0.44 to −0.49).

### 3.2 Phylogenetic relationships among the *T. turgidum* subspecies

**FIGURE 4:**
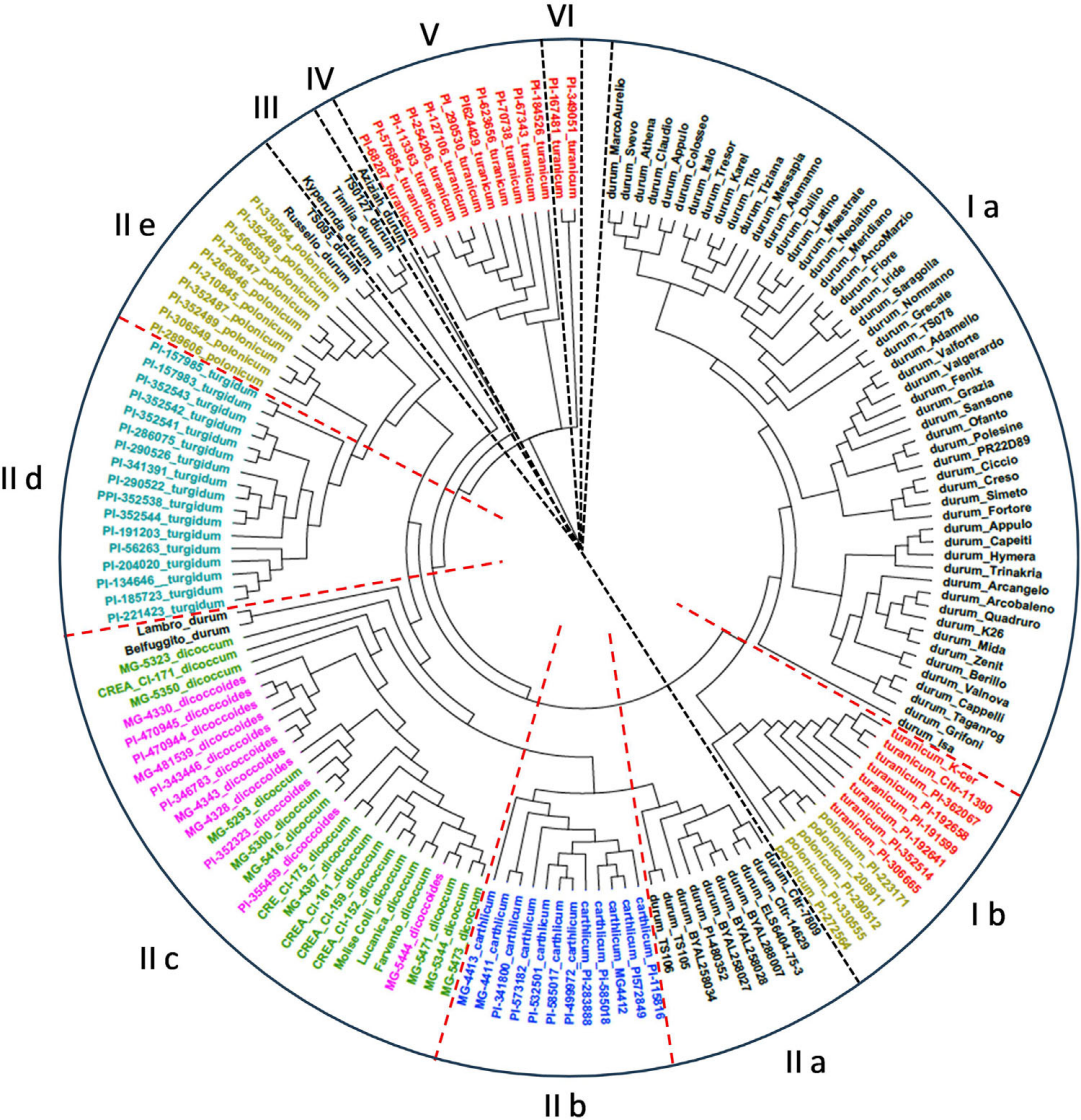
Neighbor‐joining tree based on the genetic distances of 165 tetraploid wheat accessions using 15,211 single nucleotide polymorphism (SNP) markers. The clades and subclades are delimitated using black and red dashed lines, respectively. Accessions of each subspecies are reported using different colors: *durum* (black), *turanicum* (red), *polonicum* (light brown), *carthlicum* (blue), *dicoccum* (green), *dicoccoides* (pink), and *turgidum* (turquoise).

To evaluate the genetic relationships among the *T. turgidum* subspecies, we constructed an NJ tree using a panel of 15,211 polymorphic SNP markers. Six clades were resolved using 50 as the dissimilarity threshold (Figure 4). Clades I and II included 147 out of 165 tetraploid accessions, while the clades III, IV, V, and VI consisted of the remaining 18 accessions. Clade I was further divided into two subclades (Ia and Ib). The subclade Ia included 54 out of 72 ssp. *durum* accessions, while subclade Ib consisted of 13 accessions of ssp. *turanicum* (eight) and ssp. *polonicum* (five), respectively. Clade II splits into five subclades corresponding to the durum Ethiopian accessions (IIa), ssp. *carthlicum* (IIb), ssp. *dicoccum* and ssp. *dicoccoides* together with the old durum cultivars Lambro e Belfuggito (IIc), ssp. *turgidum* (IId), and ssp. *polonicum* with the two durum landraces Russello and TS95 (IIe), respectively. Clades III and IV consisted of durum landraces, while clades V and VI comprised ssp. *turanicum* accessions.

### 3.3 QTL mapping

A total of 39 QTL significant at last in 2 years and across environments (BLUE values) for the five grain‐related traits were identified (Table 2; Table S6). The MTA findings for TKW, AREA, GL, GW, and ASPECT across years were reported as Manhattan and *Q*–*Q* plots of *p*‐values which were reported in Figure S1. Environment‐specific QTL were not shown, as only the stable QTL detected in multi‐environments are useful in marker‐assisted breeding programs.

**TABLE 2:** Quantitative trait loci (QTL) for 1000‐kernel weight (TKW), area (AREA), grain aspect (ASPECT), grain length (GL), and grain width (GW) detected in a tetraploid wheat collection in at least 2 and across years by genome‐wide association study (GWAS).

						Mean (BLUE)			
QTL	Closest marker	Closest marker ID	Chromosome	Genetic position (cM)	Physical position (Mbp)	LOD	PVE (%)	Additive effect	Trait
*QAea.mgb‐1A.1*	CAP11_c65_237	IWB13009	1A	16.5	11.7	3.5	15.8	−3.17	AREA
*QGL.mgb‐1A.1*	CAP11_c65_237	IWB13009	1A	16.5	11.7	3.5	15.9	−1.03	GL
*QGL.mgb‐1A.2*	Tdurum_contig54929_517	IWB72412	1A	48.1	313.9	4.0	19.3	0.66	GL
*QAsp.mgb‐1A*	IACX872	IWB36322	1A	49.0	–	3.7	17.6	−0.36	ASPECT
*QAea.mgb‐1A.2*	Kukri_rep_c101316_375	IWB48626	1A	50.4	357.7	4.1	19.4	−2.43	AREA
*QGL.mgb‐1A.3*	Tdurum_contig51167_390	IWB72160	1A	120.0	545.6	3.3	15.1	0.68	GL
*QGL.mgb‐1B*	Tdurum_contig68228_206	IWB73093	1B	53.9	449.3	3.3	14.8	0.76	GL
*QAsp.mgb‐1B*	RAC875_s105188_92	IWB63392	1B	54.1	450.0	3.6	21.4	−0.20	ASPECT
*QTKW.mgb‐1B*	Kukri_c5335_2165	IWB46333	1B	57.6	473.8	3.7	18.1	−8.71	TKW
*QAea.mgb‐1B.1*	Kukri_c5335_2165	IWB46333	1B	57.6	473.8	4.3	21.5	−2.94	AREA
*QAea.mgb‐1B.2*	BS00096719_51	IWB11968	1B	79.2	543.5	3.6	16.9	1.79	AREA
*QAsp.mgb‐2A.1*	Ra_c37244_428	IWB51951	2A	66.3	57.9	3.7	21.2	−0.69	ASPECT
*QAsp.mgb‐2A.2*	Tdurum_contig44687_464	IWB71534	2A	107.8	398.0	3.7	17.4	−0.37	ASPECT
*QGW.mgb‐2B*	Kukri_c1175_873	IWB40673	2B	10.0	10.4	3.6	16.7	0.14	GW
*QAsp.mgb‐2B*	BS00105409_51	IWB12242	2B	131.2	–	3.3	14.7	−0.37	ASPECT
*QGL.mgb‐2B.1*	BS00047891_51	IWB8480	2B	131.2	–	3.2	14.4	−0.92	GL
*QGL.mgb‐2B.2*	BobWhite_c852_630	IWB4485	2B	153.8	727.9	3.3	15.2	−0.72	GL
*QAsp.mgb‐3B*	Ku_c23207_988	IWB38850	3B	46.2	62.3	3.4	19.8	0.28	ASPECT
*QTKW.mgb‐3B.1*	Kukri_c66862_96	IWB47344	3B	148.4	756.4	4.6	22.9	−8.03	TKW
*QAea.mgb‐3B.1*	Kukri_c66862_96	IWB47344	3B	148.4	756.4	4.2	20.0	−2.32	AREA
*QGL.mgb‐3B.1*	Excalibur_c36725_96	IWB25749	3B	149.4	755.7	3.4	15.6	−0.59	GL
*QTKW.mgb‐3B.2*	Kukri_rep_c110544_497	IWB49458	3B	204.7	367.9	3.3	15.1	6.71	TKW
*QAea.mgb‐3B.2*	RAC875_c2106_882	IWB55121	3B	205.1	827.1	3.2	14.3	1.99	AREA
*QGL.mgb‐3B.2*	RAC875_c2106_882	IWB55121	3B	205.1	827.1	3.1	13.8	0.63	GL
*QTKW.mgb‐4B*	Excalibur_c26244_178	IWB24422	4B	51.8	402.3	3.5	16.9	6.27	TKW
*QAea.mgb‐4B*	Excalibur_c26244_178	IWB24422	4B	51.8	402.3	3.4	16.1	1.88	AREA
*QAsp.mgb‐5A.1*	CAP12_rep_c5753_98	IWB13629	5A	47.5	118.1	4.2	22.4	0.37	ASPECT
*QGL.mgb‐5A*	CAP12_rep_c5753_98	IWB13629	5A	47.5	118.1	4.2	21.8	−0.24	GW
*QAsp.mgb‐5A.2*	Tdurum_contig10587_601	IWB66619	5A	151.4	560.5	3.9	18.2	0.24	ASPECT
*QAsp.mgb‐5B*	CAP11_c919_204	IWB13083	5B	48.9	385.9	3.1	14.4	−0.47	ASPECT
*QAea.mgb‐6B*	IAAV1711	IWB34432	6B	31.3	28.7	3.5	16.1	−1.69	AREA
*QGL.mgb‐6B*	IAAV1711	IWB34432	6B	31.3	28.7	4.1	19.2	−0.60	GL
*QAsp.mgb‐7A.1*	BobWhite_c6193_298	IWB4104	7A	89.6	111.5	3.4	16.4	−0.21	ASPECT
*QAsp.mgb‐7A.2*	IAAV8081	IWB35428	7A	130.9	569.3	3.0	13.6	0.18	ASPECT
*QAsp.mgb‐7A.3*	BS00023027_51	IWB7382	7A	167.8	–	3.5	20.2	−0.37	ASPECT
*QTKW.mgb‐7A*	GENE‐4528_1252	IWB33997	7A	189.5	703.1	3.8	18.1	−6.97	TKW
*QAsp.mgb‐7B.1*	Ku_c1839_202	IWB38649	7B	63.6	139.9	3.2	19.1	−0.23	ASPECT
*QAsp.mgb‐7B.2*	Excalibur_rep_c111629_239	IWB30544	7B	98.9	538.3	3.2	14.9	0.31	ASPECT
*QAea.mgb‐7B*	Tdurum_contig46338_2305	IWB71671	7B	206.3	713.7	3.0	13.4	1.90	AREA

*Note*: Genetic (cM) and physical positions (Mbp) and additive effects and phenotypic variance explained (PVE) across years are reported. Genetic position is reported according to the durum wheat consensus map (Maccaferri et al., 2015). Physical position is reported according to the durum wheat genome sequence (Maccaferri et al., 2019). PVE (%) = Percentage of phenotypic variation explained by the single nucleotide polymorphism (SNP) marker.

Abbreviations: BLUE, best linear unbiased estimator; LOD, logarithm of the odds.

Five QTL were detected for TKW on chromosomes 1B, 3B (two QTL), 4B, and 7A, individually accounting from 15.1% to 22.9% of the phenotypic variance explained (PVE) and consistent at −log10(*p*) ranging from 3.0 to 5.0.

Nine QTL were identified for AREA on chromosomes 1A (two QTL), 1B (two QTL), 3B (two QTL), 4B, 6B, and 7B. The *QAea.mgb‐1A.1* was significant in three environments and across environments accounting for 15.8% of the phenotypic variance and with an additive effect estimated of 3.17 mm^2^. Nine QTL were detected for GL on chromosomes 1A (three QTL), 1B, 2B (two QTL), 3B (two QTL), and 6B. Interestingly, six QTL were declared in three environments and BLUE mean individually accounting from 15.1% to 19.3% of the PVE and with an additive effect ranging from 0.6 to 1.0 mm. Two QTL were found for GW on chromosomes 2B and 5A, with the −log10(*p*) of 3.1 and 4.2, respectively. Fourteen QTL were identified for ASPECT on chromosomes 1A, 1B, 2A (two QTL), 2B, 3B, 5A (two QTL), 5B, 7A (three QTL), and 7B (two QTL), eight of which were declared in three environments and BLUE mean accounting from 14.7% to 22.4% of the PVE and with additive effects comprised between 0.20 and 0.37.

**FIGURE 5:**
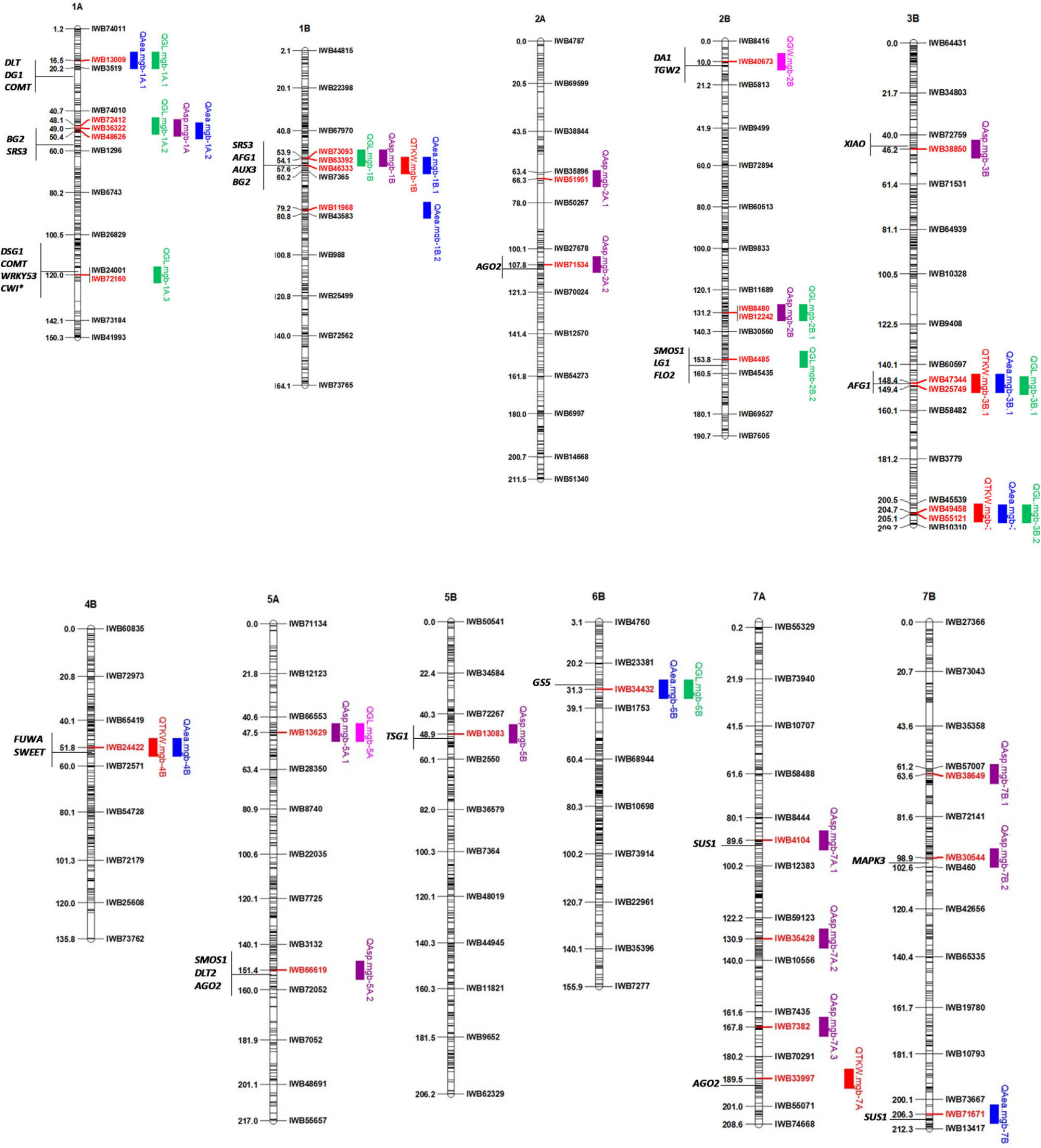
Schematic representation of A and B genome chromosomes of the durum consensus linkage map (Maccaferri et al., 2015) with map positions of quantitative trait loci (QTL) and candidate genes for 1000‐kernel weight (TKW), grain length (GL), grain width (GW), grain area (AREA), and grain aspect (ASPECT). Each chromosome map is represented by the first and the last single nucleotide polymorphism (SNP) marker and by an SNP marker every about 20 cM. Markers are indicated on the right side, and cM distances are shown on the left side of the bar. QTL are represented by bars on the right of each chromosome bar. The closest SNP marker to each QTL is indicated in red. Candidate genes potentially affecting TKW, GL, GW, AREA, and ASPECT mapping within the confidence interval of identified QTL are reported in black on the left of the chromosome bars.

Twenty‐three out of 39 QTL were grouped in nine QTL cluster regions localized on seven chromosomes (Figure 5). The QTL cluster localized on chromosome 1B included four QTL (*QGL.mgb‐1B*, *QAe.mgb‐1B.1*, *QAsp.mgb‐1B*, and *QTKW.mgb‐1B*). Three QTL clusters localized on 1A and 3B (two clusters), comprising three QTL each, and five QTL clusters included two QTL. Interestingly, among the five QTL for TKW, three QTL (*QTKW.mgb‐1B*, *QTKW.mgb‐3B.1*, *QTKW.mgb‐3B.2*) co‐located with QTL for GL and AREA on chromosomes 1B and 3B (in both the QTL clusters), respectively. In addition, three additional QTL for GL (*QGL.mgb‐1A.1*, *QGL.mgb‐1A.2*, *and QGL.mgb‐6B*) co‐located with QTL for AREA on chromosomes 1A (in both the QTL clusters) and 6B. Ten QTL for ASPECT mapped outside the QTL cluster regions, suggesting a different genetic control from the other grain traits.

### 3.4 Candidate genes underpinning grain‐related traits

To identify candidate genes for the detected QTL, we first estimated the extent of LD decay using the 12,778 polymorphic SNPs mapped in the durum reference genome (Maccaferri et al., 2019). The LD decay plot (*r* ^2^ vs. physical distance) indicated a decline in LD with increasing distance, decaying to *r* ^2^ = half decay distance at 1.77 Mbp (Figure S2). This value defined the physical interval (±1.77 Mbp) within which we searched for candidate genes. Next, we projected the closest markers associated with TKW and grain‐related traits onto the reference durum wheat cv. Svevo genome. This allowed us to pinpoint the candidate gene locations. In cases where the closest marker was not physically mapped on the Svevo genome, the closest and comigrating one in the consensus genetic map (Maccaferri et al., 2015) mapped on the reference genome was considered to delimit the physical CI. This approach revealed dozens of genes within the CI of each QTL. The total number of genes across all intervals exceeded 1000, ranging from four genes for *QAea.mgb‐4B* to 69 genes for *QAea.mgb‐1A.1*, *QGL.mgb‐1A.1*, and *QGW.mgb‐2B* (data not shown). To refine our candidate list, we prioritized genes and gene families with a known association with grain‐related traits in durum wheat from previous studies. Additionally, we considered high‐confidence genes for grain size and weight in rice, wheat, and barley, as identified and reported in the reviews by Gasparis and Miłoszewski (2023), Gao et al. (2023), and Li and Yang (2017). Our analysis identified candidate genes within six out of nine QTL clusters and nine out of 16 individual QTL intervals. These genes participate in various metabolic processes, including phytohormone signaling, sugar transport and grain filling, mitogen‐activated protein kinases (MAPK) signaling, and transcriptional regulation.

Durum wheat orthologous (and eventually their paralogues) of rice and bread wheat candidate genes involved in grain size‐related trait control were identified and reported in Table 3.

**TABLE 3:** Candidate genes located within the confidence intervals of quantitative trait loci (QTL) for 1000‐kernel weight (TKW), area (AREA), grain length (GL), grain width (GW), and grain aspect (ASPECT) detected in the tetraploid wheat collection.

QTL	Candidate gene	*Oryza sativa* gene	*Triticum aestivum* gene	Durum wheat orthologue	Durum wheat paralogues (within QTL intervals)	Physical position^a^ (Mbp)	Functional annotation	Metabolic process	References
***QAea.mgb‐1A.1/QGL.mgb‐1A.1***	*DLT*	*Os06g0127800*	*TraesCS4A02G430600 TraesCS7A02G059000 TraesCS7D02G053600*	*TRITD4Av1G248000 TRITD7Av1G014330*	*TRITD1Av1G005620*	11,504 (0.19)	Scarecrow transcription factor family protein	Phytohormone signaling	Tong et al. (2012) and Niu et al. (2022)
					*TRITD1Av1G005630*	11,509 (0.18)	Scarecrow transcription factor family protein	Phytohormone signaling	Tong et al. (2012) and Niu et al. (2022)
					*TRITD1Av1G005650*	11,529 (0.16)	Scarecrow transcription factor family protein	Phytohormone signaling	Tong et al. (2012) and Niu et al. (2022)
					*TRITD1Av1G005660*	11,535 (0.16)	Scarecrow transcription factor family protein	Phytohormone signaling	Tong et al. (2012) and Niu et al. (2022)
					*TRITD1Av1G005670*	11,550 (0.14)	Scarecrow transcription factor family protein	Phytohormone signaling	Tong et al. (2012) and Niu et al. (2022)
	*DG1*	*Os03g0229500*	*TraesCS4A02G059200 TraesCS4B02G234800 TraesCS4D02G236000*	*TRITD4Av1G027240 TRITD4Bv1G142390*	*TRITD1Av1G005730*	11,696 (4.8)^b^	Protein DETOXIFICATION	Sugar transport and grain filing	Nagasawa et al. (2013) and R. Xu et al. (2015)
	*COMT*	*Os08g0157500*	*TraesCS6A02G005600 TraesCS6B02G011100 TraesCS6D02G008200*	*TRITD6Av1G001100*	*TRITD1Av1G006350*	13,284 (1.59)	*O*‐methyltransferase	Grain size	Huangfu et al. (2022)
					*TRITD1Av1G006370*	13,299 (1.61)	*O*‐methyltransferase	Grain size	Huangfu et al. (2022)
					*TRITD1Av1G006380*	13,332 (1.64)	*O*‐methyltransferase family protein	Grain size	Huangfu et al. (2022)
***QGL.mgb‐1A.2/*** *QAea.mgb‐1A.2/ QAsp.mgb‐1A*	*BG2* (*or GE*)	*Os07g0603700*	*TraesCS2A02G175700 TraesCS2B02G201900 TraesCS2D02G183000*	*TRITD2Av1G059220 TRITD2Bv1G069570*	*TRITD1Av1G115360 TRITD1Av1G116000*	313,108 (0.81)	Cytochrome P450 family protein	Grain size	Nagasawa et al. (2013) and R. Xu et al. (2015)
	*SRS3*	*Os05g0154700*	*TraesCS1A02G095000 TraesCS1B02G123200 TraesCS1D02G104200*	*TRITD1Av1G038700 TRITD1Bv1G051340*	*TRITD1Av1G115730*	314,136 (0.25)	Kinesin‐like protein	Grain size	Deng et al. (2015) and Kitagawa et al. (2010)
*QGL.mgb‐1A.3*	*DSG1*	*Os06g0154500*	*TraesCS7A02G111300 TraesCS7B02G009200 TraesCS7D02G106400*	*TRITD7Av1G032080 TRITD7Bv1G002620*	*TRITD1Av1G209190*	545,579 (0.25)^b^	Mitogen‐activated protein kinase pseudo true	MAPK signaling	S. Liu et al. (2015) and F. Xu et al. (2018)
	*COMT*	*Os08g0157500*	*TraesCS6A02G005600 TraesCS6B02G011100 TraesCS6D02G008200*	*TRITD6Av1G001100*	*TRITD1Av1G209410*	545,887 (0.31)	*O*‐methyltransferase	Grain size	Huangfu et al. (2022)
	*WRKY53*	*Os05g0343400*	*TraesCS1A02G070400 TraesCS1B02G088900 TraesCS1D02G072900*	*TRITD1Av1G023780 TRITD1Bv1G030900*	*TRITD1Av1G209580*	546,422 (0.84)	WRKY transcription factor	MAPK signaling	Tian et al. (2021)
	*CWI* ^c^		*TraesCS2A03G0736600*	*TRITD2Av1G179620*	*TRITD1Av1G211850*	550,256 (4.68)	Starch synthase		D. Ma et al. (2012)
***QGL.mgb‐1B/QTKW.mgb‐1B/*** *QAsp.mgb‐1B/QAea.mgb‐1B.1*	*SRS3*	*Os05g0154700*	*TraesCS1A02G095000 TraesCS1B02G123200 TraesCS1D02G104200*	*TRITD1Av1G038700 TRITD1Bv1G051340*	*TRITD1Bv1G145640*	450,216 (0.91)	Kinesin‐like protein	Grain size	Deng et al. (2015) and Kitagawa et al. (2010)
	*AFG1*	*Os02g0682200*	*TraesCS6A02G259000 TraesCS6B02G286400 TraesCS6D02G240200*	*TRITD6Av1G165940 TRITD6Bv1G151090*	*TRITD1Bv1G152090 TRITD1Bv1G152940*	473,430 (0.40)	AGAMOUS‐like MADS‐box transcription factor	Transcriptional regulation	Yu et al. (2020)
	*AUX3*	*Os05g0447200*	*TraesCS1A02G278400 TraesCS1B02G287300 TraesCS1D02G277600*	*TRITD1Av1G173000 TRITD1Bv1G158190*	*TRITD1Bv1G152980*	475,571 (1.74)	Amino acid permease	Phytohormone signaling	Qiao et al. (2021)
	*BG2* (*or GE*)	*Os07g0603700*	*TraesCS2A02G175700 TraesCS2B02G201900 TraesCS2D02G183000*	*TRITD2Av1G059220 TRITD2Bv1G069570*	*TRITD1Bv1G152990*	475,580 (1.75)	Cytochrome P450	Grain size	Nagasawa et al. (2013) and R. Xu et al. (2015)
*QAsp.mgb‐2A.2*	*AGO2*	*Os04g0615700*	*TraesCS2A02G419900 TraesCS2B02G439000 TraesCS2D02G417000*	*TRITD2Av1G245870*	*TRITD2Av1G144080*	397,969 (0.07)^b^	Argonaute	Phytohormone signaling	Yin et al. (2020)
*QGW.mgb‐2B*	*DA1*		*TraesCS2B03G0048000*	*TRITD2Bv1G004840*		9,971 (0.38)	Ubiquitin–proteasome pathway	Ubiquitin–proteasome system	H. Liu et al. (2020)
	*TGW2*	*Os02g0763000*	*TraesCS6A02G321000 TraesCS6B02G351700 TraesCS6D02G300700*	*TRITD6Av1G195110 TRITD6Bv1G188160*	*TRITD2Bv1G004880*	10,015 (0.34)	Protein PLANT CADMIUM RESISTANCE 2	Grain size	Ruan et al. (2020)
					*TRITD2Bv1G004890*	10,022 (0.33)	Protein PLANT CADMIUM RESISTANCE 2	Grain size	Ruan et al. (2020)
					*TRITD2Bv1G004900*	10,039 (0.31)	Protein PLANT CADMIUM RESISTANCE 2	Grain size	Ruan et al. (2020)
*QGL.mgb‐2B.2*	*SMOS1*	*Os05g0389000*	*TraesCS1A02G242800 TraesCS1B02G254300 TraesCS1D02G242800*	*TRITD1Av1G156400 TRITD1Bv1G142930*	*TRITD2Bv1G240180*	727,457 (0.43)	RING/U‐box superfamily protein	Transcriptional regulation	Aya et al. (2014)
	*LG1*	*Os02g0244300*	*TraesCS6A02G192600 TraesCS6B02G231700 TraesCS6D02G179700*	*TRITD6Av1G097330 TRITD6Bv1G107260*	*TRITD2Bv1G240530*	728,350 (0.46)	Ubiquitin carboxyl‐terminal hydrolase	Ubiquitin–proteasome system	Shi et al. (2019)
	*Flo2*		*TraesCS2A03G1201700*	*TRITD2Bv1G240770*		728,957 (1.07)	Phytohormone signalings		Sajjad et al. (2017) and Wu et al. (2015)
*QAsp.mgb‐3B*	*XIAO*	*Os04g0576900*	*TraesCS2A02G397200 TraesCS2B02G415500 TraesCS2D02G395000*	*TRITD2Av1G234870 TRITD2Bv1G196940*	*TRITD3Bv1G023680*	61,497 (0.81)	Leucine‐rich repeat receptor‐like protein kinase	Phytohormone signaling	Jiang et al. (2012)
***QAea.mgb‐3B.2/QGL.mgb‐3B.2/QTKW.mgb‐3B.2***	*AFG1*	*Os02g0682200*	*TraesCS6A02G259000 TraesCS6B02G286400 TraesCS6D02G240200*	*TRITD6Av1G165940 TRITD6Bv1G151090*	*TRITD3Bv1G278870*	82,587 (1.27)	Transcription factor MADS‐box	Transcriptional regulation	Yu et al. (2020)
***QTKW.mgb‐4B/*** *QAea.mgb‐4B*	*FUWA*	*Os02g0234200*	*TraesCS6A02G194600 TraesCS6B02G235400 TraesCS6D02G181800*	*TRITD6Av1G100490 TRITD6Bv1G115800*	*TRITD4Bv1G113840*	400,777 (1.52)	NHL repeat‐containing protein 2	Grain size	Chen et al. (2015)
	*SWEET4*	*Os02g0301100*	*TraesCS6A02G218800 TraesCS6B02G248300 TraesCS6D02G201900*	*TRITD6Av1G137660 TRITD6Bv1G129880*	*TRITD4Bv1G114550*	403,292 (1.00)	Bidirectional sugar transporter SWEET	Sugar transport and grain filing	Sosso et al. (2015)
*QAsp.mgb‐5A.2*	*SMOS1*	*Os05g0389000*	*TraesCS1A02G242800 TraesCS1B02G254300 TraesCS1D02G242800*	*TRITD1Av1G156400 TRITD1Bv1G142930*	*TRITD5Av1G208780*	558,897 (1.62)	AP2‐like ethylene‐responsive transcription factor	Transcriptional regulation	Aya et al. (2014)
	*DLT2*	*Os03g0723000*	*TraesCS4A02G260600 TraesCS4B02G054000 TraesCS4D02G054000*	*TRITD4Av1G189680 TRITD4Bv1G016910*	*TRITD5Av1G209780*	560,892 (0.37)	GRAS transcription factor	Phytohormone signaling	Zou et al. (2023)
	*AGO2*	*Os04g0615700*	*TraesCS2A02G419900 TraesCS2B02G439000 TraesCS2D02G417000*	*TRITD2Av1G245870*	*TRITD5Av1G209870*	561,201 (0.68)	Argonaute protein	Phytohormone signaling	Yin et al. (2020)
*QAsp.mgb‐5B*	*TSG1*	*Os01g0169800*	*TraesCS3A02G093000 TraesCS3B02G108200 TraesCS3D02G093300*	*TRITD3Av1G027200 TRITD3Bv1G031000*	*TRITD5Bv1G129760*	386,439 (0.54)	Aminotransferase	Phytohormone signaling	Guo et al. (2020)
***QAea.mgb‐6B/QGL.mgb‐6B***	*GS5* ^c^		*TraesCS6A02G220200*	*TRITD6Bv1G136770*	*TRITD6Bv1G010110*	26,879 (1.80)	Carboxypeptidase	Phytohormone signaling	L. Ma et al. (2016)
*QAsp.mgb‐7A.1*	*Sus1*		*TraesCS7A03G0375000*	*TRITD7Av1G050690*		111,462 (0.05)	Starch synthase	Starch synthesis	Hou et al. (2014)
*QTKW.mgb‐7A*	*AGO2*	*Os04g0615700*	*TraesCS2A02G419900 TraesCS2B02G439000 TraesCS2D02G417000*	*TRITD2Av1G245870*	*TRITD7Av1G270890*	704,255 (1.11)	Argonaute	Phytohormone signaling	Yin et al. (2020)
				*TRITD2Av1G245870*	*TRITD7Av1G271020*	704,711 (1.57)	Argonaute	Phytohormone signaling	Yin et al. (2020)
				*TRITD2Av1G245870*	*TRITD7Av1G271030*	704,713 (1.57)	Argonaute	Phytohormone signaling	Yin et al. (2020)
*QAsp.mgb‐7B.2*	*MPK3*		*TraesCS4D02G198600*	*TRITD4Av1G049530 TRITD4Bv1G124550*	*TRITD7Bv1G170250*	538,730 (0.43)	Mitogen‐activated protein kinase	MAPK signaling	Y. Liu et al. (2022)
*QAea.mgb‐7B*	*Sus1*		*TraesCS7A03G0375000*	*TRITD7Bv1G024970*	*TRITD7Bv1G231510 *	715,507 (1.76)	Starch synthase	Starch synthesis	Hou et al. (2014)

*Note*: Orthologous genes in *Oryza sativa* and *Triticum aestivum* are reported based on the literature data assessing their role in TKW and grain size traits. Physical position, functional annotation, and metabolic processes of candidate genes identified in durum wheat are reported. Bold italics indicate the QTL belonging to a cluster surrounding the reported genes.

^a^ Physical distance (Mbp) of the candidate gene from the closest marker is reported in brackets.

^b^ Physical distance is expressed in kbp.

^c^ Outside the QTL confidence interval but with a significant role in grain‐related traits.

Specifically, in the QTL cluster on the short arm of chromosome 1A including *QAea.mgb‐1A.1* and *QGL.mgb‐1A.1*, nine genes were found, three annotated as caffeic acid *O*‐methyltransferase (*COMT*), one defective grain‐filling 1 (*DG1*), and five dwarf and low tillering (*DLT*), all known to be affecting grain size and width (Huangfu et al., 2022; Niu et al., 2022; Tong et al., 2012). In the other QTL cluster located on 1AS (comprising *QGL.mgb‐1A.2*, *QAea.mgb‐1A.2*, and *QAsp.mgb‐1A*), two *BIG GRAIN 2* (*BG2*, also named *GIANT EMBRYO*, *GE*) and the small round seed 3 (*SRS3*) genes were identified that were involved in grain size. The QTL cluster on chromosome 1B harbored five genes: two abnormal flower and grain1 (*AFG1*), an AGAMOUS‐like MADS‐box transcription factor involved in phytohormone signaling (*AUX3*), a *BG2*, and an *SRS3* gene. One more *AFG1* gene was found in the QTL region of chromosome 3B including *QAea.mgb‐3B.2*, *QGL.mgb‐3B.2*, and *QTKW.mgb‐3B.2*. Another interesting region was the QTL cluster located on chromosome 4B where two noteworthy genes were found: a *FUWA* gene and a sugar transporter *SWEET*. A carboxypeptidase (*GS5*) was found in the cluster on chromosome 6B.

Further candidate genes were found in QTL physical regions outside QTL clusters. Five *ARGONAUTE 2* (*AGO2*) genes involved in phytohormone signaling and affecting grain size were found in the physical interval of *QAsp.mgb‐2A.2*, *QAsp.mgb‐5A.2*, and *QTKW.mgb‐7A* (three genes). The other two genes were found in *QAsp.mgb‐5A.2*, small organ size 1 (*SMOS1*) and dwarf and low tillering 2 (*DLT2*). An additional *SMOS1* gene was found in *QGL.mgb‐2b.2*, along with large grain 1 (*LG1*) and the floury endosperm 2 (*FLO2*) gene. Furthermore, three tandem cell number regulator 1 (*TGW2*) genes were found in the CI of *QGW.mgb‐2*B, along with the ubiquitin receptor (*DA1*). Genes involved in MAPK signaling were also found, such as a dwarf and small grain 1 (*DSG1*) and a WRKY53 transcription factor, within the *QGL.mgb‐1A.3* physical interval, and a mitogen‐activated protein kinases 3 (*MPK3*) in the *QAsp.mgb‐7B.2* region. Noteworthy, a cell wall invertase (*CWI*) was found a few Mbp downstream the interval of *QGL.mgb‐1A.3*. A *XIAO* gene, which encodes another leucine‐rich repeat receptor‐like protein kinase protein that positively influences GL was found in *QAsp.mgb‐3B* and a tillering and small grain 1 (*TSG1*) in controlling auxin synthesis in *QAsp.mgb‐5B*. Interestingly, two homoeologues genes encoding for sucrose synthase 1 (*Sus1*) were located within the intervals of *QAsp.mgb‐7A.1* and *QAea.mgb‐7B*, respectively.

### 3.5 Gene ontology enrichment analysis

In order to gain insight into the overrepresented biological processes, molecular functions (MFs), and cellular components (CC) associated to all genes underlying each identified QTL, a GO enrichment analysis was performed.

Six GO terms of “cellular components” and four “molecular function” were shared among the grain‐related traits (Table S7). In particular, five GO terms were overrepresented (with a *p*‐value adjusted ≥ 0.001) for both GL and AREA: “*O*‐methyltransferase activity” (MF, GO:0008171), “Ndc80 complex” (CC, GO:0031262), “outer kinetochore” (CC, GO:0000940), “kinetochore” (CC, GO:0000776), and “AP‐3 adaptor complex” (CC, GO:0000779) (Table S7). Four GO terms were commonly shared by GW and ASPECT: “heme binding” (MF, GO:0020037), “tetrapyrrole binding” (MF, GO:0046906), “chloroplast thylakoid lumen” (CC, GO:0009543), and “plastid thylakoid lumen” (CC, GO:0031978). Finally, “alternative oxidase activity” (MF, GO:0009916) was found shared between GL and ASPECT.

The GO analysis for TKW identified the “DNA binding” (MF, GO:0003677) comprising three candidate genes within the physical regions of TKW QTL, *TRITD1Bv1G152090*, *TRITD1Bv1G152940*, and *TRITD1Bv1G152980*, respectively, coding for AGAMOUS‐like MADS‐box transcription factor, PISTILLATA‐like MADS‐box transcription factor, and amino acid permease (Table S7). Among the GO terms identified for AREA, the one with the highest overrepresentation, “*O*‐methyltransferase activity” (MF, GO:0008171), included three candidate genes (*TRITD1Av1G006350*, *TRITD1Av1G006370*, and *TRITD1Av1G006380*), localized on chromosome 1A affecting GL and AREA. The GO term GO:0004185, corresponding to “serine‐type carboxypeptidase activity,” included the candidate gene *TRITD6Bv1G010110* coding for a carboxypeptidase (Grain Size 5).

In addition, the GO enrichment analysis underlined several other GO terms related to methyltransferase activity, ubiquitination processes, and post‐translational protein regulation for candidate genes retrieved in GL QTL physical regions. Noteworthy, among the identified cellular compartment GO terms highlighted by the analysis of physical regions surrounding GW, four of them were referred to as chloroplast/thylakoid lumen. Finally, several GO terms related to biological processes concerning oxidation reactions were reported for the candidate genes in the physical regions of QTL for ASPECT.

## 4 DISCUSSION

Grain size affects TKW, one of the major yield components used as a target in wheat breeding programs. Assessment of grain size and TKW in wheat germplasm combined with QTL detection and candidate genes identification can assist grain yield improvement. Grain size is tightly underpinned by grain morphology, including AREA, GL, GW, and ASPECT, which are easily determined by digital imaging analysis. In this study, the grain‐related traits are highly heritable (Table 1; Figure 3) and significantly positively correlated to TKW, suggesting that grain weight is strongly determined by grain size.

### 4.1 Grain size and weight variation in tetraploid wheat germplasm

Phenotypic analysis of 165 tetraploid wheat accessions showed highly significant differences (*p *< 0.001) in TKW and grain‐related traits evaluated in three field trials. A wide variation was found for TKW, AREA, and GL among the *T*. *turgidum* subspecies, ranging from the highest values for the ssp. *turanicum* accessions to the lowest ones for the ssp. *carthlicum* accessions. The ssp. *turgidum* showed higher GW values compared to the wild emmer ssp. *dicoccoides* accessions, although this trait was the least variable in the whole tetraploid wheat collection. As expected, the phenotypic variation observed for GL and GW affected the ASPECT. Indeed, the ssp. *dicoccoides* showed higher ASPECT values as a result of its lower GW, while the ssp. *carthlicum* showed lower ASPECT values as a consequence of its lower GL. The PCA captured the grain size variation as shown by the first two PCs explaining over 95% of the total variation. Interestingly, the ssp. *turanicum* and ssp. *carthlicum* corresponded to the two extreme groups along PC1 suggesting that the TKW, AREA, and GL are the main explanatory determinants. On the other hand, ssp. *turgidum* and ssp. *dicoccoides* represented the two extreme groups along PC2 indicating the GW as the main explanatory factor. Therefore, PC1 captured variation in grain shape primarily through changes in GL, AREA, and TKW, while PC2 captured variation in grain size through changes in GW and ASPECT. Our result indicated that grain size increased through independent changes in GL or GW from wild to domesticated subspecies. Previously, Gegas et al. (2010) found that grain size and shape were independent traits under the control of distinct genetic elements in different mapping populations of bread wheat. They suggested that wheat domestication resulted in a change from small seeds with a long and thin shape to larger seeds. The present study found strong grain size and shape differences between the ssp. *carthlicum* and the other *T. turgidum* subspecies. Taranto et al. (2021) highlighted that the ssp. *carthlicum* accessions formed a distinctive group from the other *T. turgidum* subspecies for their high polyphenol oxidase activity. The gliadin profile of ssp. *carthlicum* is similar to that of hexaploid wheat, which led to its initial classification as a hexaploid species (Bushuk & Kerber, 1978). Takumi and Morimoto (2015) suggested that the ssp. *carthlicum* could be derived from interploidy hybridization between tetraploid and hexaploid wheat species. Our results on grain size and TKW seem to support this hypothesis.

The durum cultivars showed less GL and GW variation than old cultivars with wild and domesticated subspecies. Therefore, the grain size variability of wild and domesticated wheat germplasm can be useful in identifying new loci or alleles involved in the control of grain shape and size traits (Gegas et al., 2010; Kumar et al., 2016). In addition, the narrow GL variation could be the result of the selection for ASPECT considering the relationship of this trait with test weight, semolina yield, and grain quality (K. Wang et al., 2021).

### 4.2 SNPs as a source to discriminate tetraploid wheat subspecies

The phylogenetic analysis based on high‐quality SNPs (Figure 4) on the whole reflects the taxonomic classification of the *T. turgidum* subspecies according to van Slageren (1994) with a few variations concerning the *ssp. turanicum* and *ssp. polonicum* accessions. Indeed, the ssp. *turanicum* accessions were clustered in the clades I, V, and VI, while the ssp. *polonicum* accessions were split into clades I and II. The presence of accessions belonging to ssp. *turanicum* and ssp. *polonicum* with durum wheat in the clade I, suggested a genetic relationship among these subspecies. Khlestkina et al. (2006) proposed that ssp. *turanicum* could be a natural hybrid between ssp. *durum* and ssp. *polonicum*. On the other hand, the ssp. *polonicum* subclade in the clade II suggested that this subspecies could be originated from the natural hybridization with other domesticated wheat such as the ssp. *turgidum* accessions. Two durum wheat (Russello and TS095) grouped together with accessions of ssp. *polonicum* in the clade II. This result could be attributed to natural hybridization between durum landraces and Polish wheat accessions. In the clades V and VI, we exclusively found accessions of ssp. *turanicum* originated from the Near East. This result suggested a low contribution of this germplasm to the durum cultivars, supporting the hypothesis of Maccaferri et al. (2019).

In the clade II, the wild emmer (ssp. *dicoccoides*) clustered with domesticated emmer (ssp. *dicoccum*), confirming the genetic relationships between these wheat subspecies. The old durum cultivars Lambro and Belfuggito grouped with wild and domesticated emmer accessions as they derived from introgression with emmer wheat (Laidò et al., 2013). Clade II also includes the ssp. *carthlicum*, ssp. *turgidum*, and the Ethiopian durum accessions. In a genetic diversity study performed on over 15,000 domesticated tetraploid wheats, the durum accessions collected in Ethiopia resulted distinct from the durum elite germplasm (Sansaloni et al., 2020), indicating the genetic diversity of Ethiopian durum germplasm. Finally, clades III and IV included durum landraces derived from Mediterranean durum germplasm.

### 4.3 QTL for grain size and weight and comparison with previous studies

The current study used a high‐density genetic map based on SNPs to identify QTL for grain weight and grain size‐related traits in a tetraploid wheat collection including seven *T. turgidum* subspecies. A total of 39 QTL were detected for the five examined grain‐related traits. Five QTL for TKW were identified on four different chromosomes (1B, 3B, 4B, and 7A) that were significant across environments and in at least two environments. Additionally, 11 QTL affecting the primary grain size traits were detected: nine for GL and two for GW (Table 2; Figure 5).

To consider the possible influence of GL and GW and their interaction on grain weight, the QTL analysis was also carried out on some grain size‐derived traits such as AREA and ASPECT. This analysis detected 14 QTL for ASPECT and nine QTL for AREA, and highlighted the overlapping of the CI of four TKW QTL with the CI of the AREA QTL, as well as the overlapping of the CI of GL QTL with that of AREA QTL, thus corroborating the results of the positive correlation between the above traits.

Based on the map position, 23 out of 39 QTL were grouped in nine clusters comprising QTL for the primary grain traits (TKW, GL, and GW) and QTL for the grain size‐derived traits (AREA and ASPECT).

Finding the same QTL (gene regions) across different wheat natural collection or biparental segregant populations would strengthen the evidence for their role and narrow down their location. However, comparing results from various studies is challenging. Researchers often use different markers to identify QTL, and there is no agreed‐upon reference point for many markers mentioned in published papers. Moreover, the genetic materials used in each study can have very different levels of genetic map detail.

For *T. turgidum* collections, these challenges can be partially addressed. Recent advancements include a high‐quality wild emmer genome sequence (Avni et al., 2017), a durum wheat Svevo genome sequence (Maccaferri et al., 2019), and a standardized durum wheat map with over 30,000 markers (Maccaferri et al., 2015). This map allows researchers to compare results across studies. In this study, we linked the SNP markers on the Svevo durum wheat genome and that allowed us to compare our findings (QTL) with previously reported QTL and to identify potentially candidate genes related to grain size and weight. We considered QTL to be potentially co‐located if the CIs overlapped, either completely or partially.

Many investigations have explored QTL/genes for grain weight (TKW) in wheat. Some studies have explored how genes for grain shape and size influence TKW, but this research has primarily focused on bread wheat, with just a few studies carried out in durum wheat (Golan et al., 2015; Russo et al., 2014). Additionally, most studies on tetraploid wheat have used low‐density simple sequence repeat‐based maps to detect and analyze QTL/genes for grain size. Only a few recent studies have used more advanced, high‐density genetic maps for durum and emmer wheat.

The grain‐related traits QTL detected in the present study were compared with previously identified QTL for the same traits, found using either linkage or association mapping, and recently mapped to specific locations on the reference genome (as described in Maccaferri et al. [2019]) and with more recent studies on tetraploid wheat (Desiderio et al., 2019; Mangini et al., 2021; Soriano et al., 2017; Sun et al., 2020; Valladares Garcia et al., 2023). This process revealed potential overlaps between our QTL clusters and previously mapped QTL.

Remarkably, most of the QTL here identified for grain weight and grain size‐related traits overlapped with known locations for genes involved in the metabolic process of grain size. In some cases, we found overlaps between our QTL and previously identified QTL for the same traits in other studies, thus corroborating the expression and detection of QTL in multi‐environmental conditions.

For example, the detected *QTKW.mgb‐1B* on chromosome 1B at 57.6 cM (physical position at 473.8 Mbp) could correspond to the QTL previously reported by Peng et al. (2003) in a mapping population derived from a cross between the ssp. *dicoccoides* and ssp. *durum*, by Soriano et al. (2017) in a Mediterranean durum wheat collection, and by Sun et al. (2020) in a worldwide collection of durum wheat germplasm. Faris et al. (2014), Mangini et al. (2018), Sun et al. (2020), and Valladares Garcia et al. (2023) reported QTL for TKW on the long arm of chromosome 3B that could coincide with *QTKW.mgb‐3B.1* mapped at 148.4 cM (756.3 Mbp). *QTKW.mgb‐4B* on chromosome 4B at 51.8 cM (402.3 Mbp) could correspond to the QTL previously reported by Peleg et al. (2011), Patil et al. (2013), and Soriano et al. (2017).

Notably, for GL, nine stable QTL were detected on five different chromosomes with PVE ranging from 13.8% to 19.3%; two of these QTL, *QGL.mgb‐1A.3* and *QGL.mgb‐6B*, identified on chromosomes 1A at 120 cM (545.6 Mbp) and 6B at 31.3 cM (28.7 Mbp), respectively, are likely to be novel as no overlapping was found with other previously reported QTL at these genomic regions.

### 4.4 Candidate genes for grain size and weight

Several studies estimated the LD decay as genetic distances (cM) in durum wheat using different classes of molecular markers (Laidò et al., 2014; Maccaferri et al., 2014; Soriano et al., 2017). The availability of a high‐density SNP map (Maccaferri et al., 2015) along with the durum reference genome (Maccaferri et al., 2019) has allowed the estimation of the LD decay as physical distance (Mb), thereby increasing its precision (Taranto et al., 2020). Therefore, the candidate gene search on the durum wheat reference genome sequence should be more accurate.

Among the candidate genes retrieved in the QTL cluster including *QAea.mgb‐1A.1* and *QGL.mgb‐1A.1* on chromosome 1A, three were noteworthy: *COMT*, *DG1*, and *DLT* genes, all known affecting GL and GW.

*COMT* belongs to the *O*‐methyltransferase (OMT) family, involved in melatonin biosynthesis (Byeon et al., 2015). Melatonin has been considered an important plant growth regulator that influences seed germination, photosynthesis, and protection against abiotic and/or biotic stress (see review Arnao and Hernández‐Ruiz, 2019). Recently, the *OsCOMT* gene was found to affect GL, GW, and TGW in rice through dual regulation of leaf senescence and vascular development, suggesting a positive role of this gene in grain yield improvement (Huangfu et al., 2022). In our study, two durum wheat paralogues *COMT* genes were found on chromosomes 1A in the QTL cluster including *QAea.mgb‐1A.1* and *QGL.mgb‐1A.1*, and in the physical region surrounding *QGL.mgb‐1A.3*, respectively. Indeed, GO enrichment analysis outlined for both AREA and especially GL several MF terms related to methyltransferase activity, thus underlying the importance of these molecular processes in the grain size expression.

*DG1* gene encodes for a multidrug and toxic compound extrusion (MATE) transporter that regulates the long‐distance leaf‐to‐caryopsis abscisic acid (ABA) leucine‐rich repeat transport. Qin et al. (2021) showed that the rice mutant *dg1* failed to accumulate leaf‐derived ABA, which activates starch biosynthesis genes, leading to the formation of incompletely filled, floury seeds with a significantly reduced TKW. They also observed the same defects in mutants of the maize *DG1* orthologous gene, suggesting a conserved function of *DG1* in cereal species. We identified in durum wheat a paralogues of orthologous rice gene *DG1*, validating the relationship of these genes with TKW and the conserved function in monocot.

*DLT* gene encodes important regulators of brassinosteroids (BRs) response. BRs affect a wide range of physiological processes including cell elongation, cell division and differentiation, flowering, seed number, and seed size. In rice, the *dlt* mutant resulted in 6.8% larger than that of its wild‐type parent (Sun et al., 2013). Niu et al. (2022) found that a *dlt* mutant showed a semi‐dwarf phenotype in BR‐deficient plants, but it was also shown that the mutation had positive impacts on GW and TKW.

Our results suggested that in wheat, *BG2* (also named GIANT EMBRYO, *GE*) and *SRS3* are two notable genes affecting grain size and TKW. Both genes were mapped on the chromosome 1A QTL cluster including *QGL.mgb‐1A.2*, *QAea.mgb‐1A.2*, and *QAsp.mgb‐1A*, and on the 1B QTL cluster. R. Xu et al. (2015) identified a rice mutant line carrying a mutation in *GE2* locus that increased GL, GW, and grain weight. This locus encodes for a CYP78A13, belongs to cytochrome P450 (CYP), and regulates the balance between embryo and endosperm size (Nagasawa et al., 2013). Kitagawa et al. (2010) observed that the cell length of grain in an *SRS3* mutant was shorter than that in the wild type. These results suggested that *SRS3* affects the cell elongation process. This gene belongs to the kinesin 13 subfamily and encodes an active microtubule depolymerase. In addition, the GO enrichment analysis identified several terms related to the kinetochore complex, confirming the crucial roles of *SRS3* for both AREA and especially GL.

Among the genes identified within the physical region surrounding *QGL.mgb‐2B.2*, a *Flo2* gene was annotated. *Flo2* is highly conserved across plant species. Its role was first defined in rice, where it was found to influence the starch synthesis‐related genes resulting positively correlated with amylose content and grain weight (She et al., 2010; Wu et al., 2015). Sajjad et al. (2017) reported that bread wheat *TaFlo2‐A1*, an ortholog of rice *OsFlo2*, plays the same role. Moreover, they found that the highly expressed haplotype *TaFlo2‐A1b* is significantly associated with high TGW.

The *AFG1* gene belongs to MADS‐box transcription factors family involved in floret formation and grain development. Yu et al. (2020) reported that an *afg1* mutant showed higher GW and lower GL compared to the wild type. Moreover, they demonstrated that the transcript levels of main grain size‐related genes (including *GE1*, *GE2*, and *GW2*), as well as other genes related to cell expansion, were altered in the *afg1* mutant, suggesting that *AFG1* controls grain size by acting as a transcriptional activator. Interestingly, we found the paralogues of these orthologous rice genes in two QTL clusters mapped on chromosomes 1B and 3B, respectively, which both showed the overlapping of GL and TKW QTL.

The QTL cluster, including *QTKW.mgb‐4B* and *QAea.mgb‐4B*, is particularly noteworthy as two crucial candidate genes were found: *FUWA* and *SWEET*. *FUWA* encodes an NHL domain‐containing protein, highly conserved in monocots, mainly expressed in the root and shoot apical meristem, as well as in inflorescences. The loss of function of *FUWA* leads to increased GW, ASPECT, and grain weight as well as decreased GL (Chen et al., 2015).

SWEETs are bidirectional sugar transporters, a highly conserved gene family, involved in several processes, such as phloem loading and grain filling, and plant–pathogen interactions (Breia et al., 2021). SWEETs may also transport phytohormones such as gibberellins (Kanno et al., 2016) and cytokinins (Radchuk et al., 2023). In the physical region surrounding *QTKW.mgb‐4B*, we found a paralogues gene to rice *SWEET4* and barley *SWEET11* orthologous. Sosso et al. (2015) experimentally validated several SWEET transporters in rice and their crucial role in proper grain filling, as their knockout resulted in defective endosperm manifested as concavities in the mature caryopses or in the formation of very thin grains. Rudchuk et al. (2023) showed that *HvSWEET11b* transporters are also crucial for barley grain development, as they are involved in grain filling and endosperm formation. Expression and tissue localizations of SWEET transporters are similar in barley and rice, as well as their ability for cytokinins transport, thus further enhancing endosperm development.

As reported by Gao et al. (2023) and Gasparis and Miłoszewski (2023), the grain size in cereals is also affected by the genes involved in the ubiquitin–proteasome pathway, which plays a critical role in seed development by ubiquitinating and degrading proteins, resulting in the enhancement or inhibition of cell proliferation.

We found a durum wheat ubiquitin receptor *DA1* within the interval of *QGW.mgb‐2B*. This receptor is a conserved component of the ubiquitin–proteasome system and negative regulator of kernel size, as it was found to limit the proliferation of maternal pericarp cells. H. Liu et al. (2020) reported that the wheat *TaDA1* also had an additive effect on *TaGW2*, an E3 RING ubiquitin ligase well‐known to be a negative regulator of grain weight.

Likewise, several genes involved in starch and sucrose metabolism pathways in wheat have important roles in controlling grain weight and size, including the cell wall invertase genes *TaCwi‐A1* and the sucrose synthase genes *TaSus1* and *TaSus2* (Hou et al., 2014; D. Ma et al., 2012; Volpicella et al., 2016).

Interestingly, two homoeologoues genes encoding for *Sus1* were indeed located within the intervals of *QAsp.mgb‐7A.1* and *QAea.mgb‐7B*, respectively, while a *CWI* was found downstream the interval of *QGL.mgb‐1A.3*, thus supporting the involvement of these genes in TGW and seed traits considered in this study, and the importance of the identified genomic regions as potential targets for future breeding programs aimed at yield improvement.

## 5 CONCLUSION

The grain yield improvement of durum wheat can be realized detecting QTL/genes for grain weight and grain size‐related traits and using the associated molecular markers in MAS programs. Our work suggest that the grain phenotyping and SNP genotyping of tetraploid wheat germplasms is a useful approach to perform GWAS and for detecting genomic regions involved in grain size and weight. A total of 39 QTL were detected for grain related traits. Some of these QTL overlap with previous reported QTL and are further strengthened by the known functions of the genes associated with the markers and their similarity to genes in other cereal species. In the QTL regions associated to grain weight and grain size‐related traits, several candidate genes that can play a strategic role in the process such as seed growth and development, cell elongation, phytohormone signaling, and sugar transporter, were identified. However, the discovery of many potential genes linked to grain‐related traits needs further investigation in durum wheat to confirm their functions, also through by the utilization of revolutionary genome editing tools like CRISPR/Cas9. Additional research is needed to validate the markers associated with QTL for grain size and weight, including fine mapping. Moreover the conversion of SNP markers to kompetitive allele‐specific PCR (KASP) should be performed before they are directly used in new breeding programs.

## AUTHOR CONTRIBUTIONS

**G. Mangini**: Conceptualization; data curation; formal analysis; resources; supervision; writing—review and editing. **D. Nigro**: Data curation; formal analysis; funding acquisition; writing—review and editing. **P. L. Curci**: Formal analysis; writing—review and editing. **R. Simeone**: Funding acquisition; project administration; resources. **A. Blanco**: Conceptualization; resources; supervision; writing—review and editing.

## CONFLICT OF INTEREST STATEMENT

The authors declare no conflicts of interest.

## Supporting information




**Supplemental Figure S1**. Manhattan and quantile–quantile plots generated using a mixed linear model (MLM) for (a) 1000‐kernel weight (TKW), (b) area (AREA), (c) grain length (GL), (d) grain width (GW), and (e) grain aspect (ASPECT) in a tetraploid wheat collection across 3 years.
The significance level (−log10(*p*) ≥ 3.0) is represented by the red dashed horizontal line. The *X*‐axis shows the position of SNPs along the 14 chromosomes, with various colors indicating distinct chromosomes. The *Y*‐axis shows the −log10(*p*) observed in each analysis.
**Supplemental Figure S2**. Linkage disequilibrium (LD) decay in the tetraploid wheat collection. Pairwise LD (*r* ^2^) values plotted versus corresponding pairwise physical distance (Mbp) of SNPs. The trend line of nonlinear regression against physical distance is given by the red line. The point at which LD is reduced to 50% of its maximum value is indicated by the green vertical line.

**Supplemental Table S1**. *Triticum turgidum* subspecies and accessions included in the tetraploid wheat collection. Pedigree, clades and sub‐clades were drawn according to the phylogenetic relationship analysis using a panel of 15,211 polymorphic SNP markers (section 3.2) are reported.
**Supplemental Table S2**. Analysis of variance and heritability (*h* ^2^_B_) of 1000‐kernel weight (TKW), area (AREA), grain length (GL), grain width (GW), and grain aspect (ASPECT) in a tetraploid wheat collection evaluated at Valenzano (Bari, Italy) over 3 years (2010, 2013 and 2014).
**Supplemental Table S3**. Combined analysis of variance and heritability (*h* ^2^_B_) of 1000‐kernel weight (TKW), area (AREA), grain length (GL), grain width (GW), and grain aspect (ASPECT) in a tetraploid wheat collection evaluated at Valenzano (Bari, Italy) for 3 years (2010, 2013 and 2014).
**Supplemental Table S4**. Pearson correlation coefficient (r) of 1000‐kernel weight (TKW), area (AREA), grain length (GL), grain width (GW), and grain aspect (ASPECT) between the three environments in a tetraploid wheat collection evaluated at Valenzano (Bari, Italy) for 3 years (2010, 2013 and 2014).
**Supplemental Table S5**. Pearson correlation coefficient (r) of 1000‐kernel weight (TKW), area (AREA), grain length (GL), grain width (GW), and grain aspect (ASPECT) in a tetraploid wheat collection evaluated at Valenzano (Bari, Italy) for 3 years (2010, 2013 and 2014).
**Supplemental Table S6**. Quantitative trait loci (QTL) for 1000‐kernel weight (TKW), area (AREA), grain aspect (ASPECT), grain length (GL), and grain width (GW) detected in a tetraploid wheat collection in single‐year and across‐years by genome‐wide association study (GWAS).
**Supplemental Table S7**. Gene ontology (GO) enrichment analysis performed in the regions flanking the closest marker to each QTL for TKW and grain traits (GL, GW, AREA, and ASPECT). Ontology: MF = molecular function, BP = biological process, and CC = cellular components.

## Data Availability

All data supporting the findings of this study are available within the paper and within its Supporting Information.
