# Correction to “Genome‐wide association study identifies QTL and candidate genes for grain size and weight in a *Triticum turgidum* collection”

**DOI:** 10.1002/tpg2.70158

**Published:** 2025-11-24

Mangini, G., Nigro, D., Curci, P. L., Simeone, R., & Blanco, A. (2025). Genome‐wide association study identifies QTL and candidate genes for grain size and weight in a *Triticum turgidum* collection. *The Plant Genome, 18*(1), e20562. https://doi.org/10.1002/tpg2.20562

The original Table 1, shown below, contained errors for the mean, standard error, minimum, maximum, and CV for ssp*. turgidum* values in AREA, GL, GW, and ASPECT.

**TABLE 1:** Mean values, standard error, range of variation (minimum and maximum), coefficient of variation (CV) and heritability (*h* ^2^_B_) of 1000‐kernel weight (TKW), area (AREA), grain length (GL), grain width (GW), and grain aspect (ASPECT) in a tetraploid wheat collection evaluated at Valenzano (Bari, Italy) for 3 years (2010, 2013, and 2014).

Trait	*T*. *turgidum* ssp.^*^	Mean	Standard error	Minimum	Maximum	CV (%)	*h* ^2^_B_
TKW (g)	Whole collection (165)	48.9	1.09	26.1	81.3	23.1	0.94
	ssp. *durum* (72)	48.5	0.75	37.0	63.5	13.0	
	Landraces and old cultivars (26)	46.2	1.48	37.0	63.5	16.4	
	Modern cultivars (46)	49.8	0.76	40.0	61.3	10.3	
	ssp. *turanicum* (21)	62.0	2.46	37.0	81.3	17.9	
	ssp*. polonicum* (15)	59.6	2.58	44.5	74.0	16.8	
	ssp. *turgidum* (17)	49.4	2.40	34.4	67.5	20.1	
	ssp. *carthlicum* (12)	31.6	1.09	28.3	38.7	11.9	
	ssp. *dicoccum* (17)	46.8	1.67	35.4	60.9	14.8	
	ssp. *dicoccoides* (11)	33.9	2.01	26.1	42.2	19.7	
AREA (mm^2^)	Whole collection	20.6	0.32	12.8	29.6	16.9	0.97
	ssp. *durum*	19.8	0.19	16.2	23.0	8.1	
	Landraces and old cultivars	19.1	0.38	16.2	23.0	10.1	
	Modern cultivars	20.1	0.19	17.8	22.3	6.3	
	ssp. *turanicum*	25.4	0.64	17.7	29.6	11.4	
	ssp*. polonicum*	24.1	0.73	19.7	27.8	11.7	
	ssp. *turgidum*	19.6	0.47	0.2	29.6	30.1	
	ssp. *carthlicum*	14.6	0.44	12.8	17.7	10.5	
	ssp. *dicoccum*	22.0	0.49	18.4	25.0	9.1	
	ssp. *dicoccoides*	19.3	0.71	15.2	22.4	12.2	
GL (mm)	Whole collection	8.1	0.11	5.6	10.7	14.1	0.98
	ssp. *durum*	7.7	0.04	6.4	8.3	4.6	
	Landraces and old cultivars	7.6	0.08	6.4	8.3	5.6	
	Modern cultivars	7.7	0.04	7.1	8.3	3.9	
	ssp. *turanicum*	9.7	0.21	7.3	10.7	9.6	
	ssp*. polonicum*	9.1	0.20	7.7	10.1	8.5	
	ssp. *turgidum*	7.5	0.18	0.0	10.7	29.9	
	ssp. *carthlicum*	6.5	0.17	5.6	7.7	9.0	
	ssp. *dicoccum*	8.8	0.10	7.9	9.5	4.5	
	ssp. *dicoccoides*	8.8	0.20	7.6	9.6	7.6	
GW (mm)	Whole collection	3.3	0.04	2.5	3.8	8.3	0.94
	ssp. *durum*	3.3	0.02	2.7	3.6	6.2	
	Landraces and old cultivars	3.2	0.05	2.7	3.6	8.2	
	Modern cultivars	3.3	0.02	3.1	3.6	4.6	
	ssp. *turanicum*	3.3	0.04	3.1	3.6	5.0	
	ssp*. polonicum*	3.4	0.04	3.2	3.7	5.1	
	ssp. *turgidum*	3.2	0.08	0.0	8.2	31.1	
	ssp. *carthlicum*	2.9	0.03	2.7	3.1	3.2	
	ssp. *dicoccum*	3.2	0.04	2.9	3.5	5.6	
	ssp. *dicoccoides*	2.8	0.07	2.5	3.2	8.4	
ASPECT	Whole collection	2.5	0.04	1.7	3.7	15.9	0.97
	ssp. *durum*	2.3	0.02	2.0	2.9	7.9	
	Landraces and old cultivars	2.4	0.05	2.0	2.9	10.6	
	Modern cultivars	2.3	0.02	2.1	2.6	5.7	
	ssp. *turanicum*	2.9	0.06	2.3	3.4	10.0	
	ssp*. polonicum*	2.7	0.05	2.3	3.0	7.9	
	ssp. *turgidum*	2.5	0.11	0.0	10.6	53.4	
	ssp. *carthlicum*	2.3	0.06	2.0	2.6	8.5	
	ssp. *dicoccum*	2.8	0.03	2.6	3.0	3.8	
	ssp. *dicoccoides*	3.2	0.09	2.6	3.7	9.5	

^*^Number of accessions of whole collection and of *T. turgidum* ssp. reported in round brackets of TKW.

The corrected values for the mean, standard error, minimum, maximum, and CV for ssp*. turgidum* in AREA, GL, GW, and ASPECT in Table 1 are shown below:

**TABLE 1:** Mean values, standard error, range of variation (minimum and maximum), coefficient of variation (CV) and heritability (*h* ^2^_B_) of 1000‐kernel weight (TKW), area (AREA), grain length (GL), grain width (GW), and grain aspect (ASPECT) in a tetraploid wheat collection evaluated at Valenzano (Bari, Italy) for 3 years (2010, 2013, and 2014).

Trait	*T*. *turgidum* ssp.^*^	Mean	Standard error	Minimum	Maximum	CV (%)	*h* ^2^_B_
TKW (g)	Whole collection (165)	48.9	1.09	26.1	81.3	23.1	0.94
	ssp. *durum* (72)	48.5	0.75	37.0	63.5	13.0	
	Landraces and old cultivars (26)	46.2	1.48	37.0	63.5	16.4	
	Modern cultivars (46)	49.8	0.76	40.0	61.3	10.3	
	ssp. *turanicum* (21)	62.0	2.46	37.0	81.3	17.9	
	ssp*. polonicum* (15)	59.6	2.58	44.5	74.0	16.8	
	ssp. *turgidum* (17)	49.4	2.40	34.4	67.5	20.1	
	ssp. *carthlicum* (12)	31.6	1.09	28.3	38.7	11.9	
	ssp. *dicoccum* (17)	46.8	1.67	35.4	60.9	14.8	
	ssp. *dicoccoides* (11)	33.9	2.01	26.1	42.2	19.7	
AREA (mm^2^)	Whole collection	20.6	0.32	12.8	29.6	16.9	0.97
	ssp. *durum*	19.8	0.19	16.2	23.0	8.1	
	Landraces and old cultivars	19.1	0.38	16.2	23.0	10.1	
	Modern cultivars	20.1	0.19	17.8	22.3	6.3	
	ssp. *turanicum*	25.4	0.64	17.7	29.6	11.4	
	ssp*. polonicum*	24.1	0.73	19.7	27.8	11.7	
	ssp. *turgidum*	18.8	0.59	14.7	23.1	12.9	
	ssp. *carthlicum*	14.6	0.44	12.8	17.7	10.5	
	ssp. *dicoccum*	22.0	0.49	18.4	25.0	9.1	
	ssp. *dicoccoides*	19.3	0.71	15.2	22.4	12.2	
GL (mm)	Whole collection	8.1	0.11	5.6	10.7	14.1	0.98
	ssp. *durum*	7.7	0.04	6.4	8.3	4.6	
	Landraces and old cultivars	7.6	0.08	6.4	8.3	5.6	
	Modern cultivars	7.7	0.04	7.1	8.3	3.9	
	ssp. *turanicum*	9.7	0.21	7.3	10.7	9.6	
	ssp*. polonicum*	9.1	0.20	7.7	10.1	8.5	
	ssp. *turgidum*	6.8	0.13	6.0	7.9	7.9	
	ssp. *carthlicum*	6.5	0.17	5.6	7.7	9.0	
	ssp. *dicoccum*	8.8	0.10	7.9	9.5	4.5	
	ssp. *dicoccoides*	8.8	0.20	7.6	9.6	7.6	
GW (mm)	Whole collection	3.3	0.04	2.5	3.8	8.3	0.94
	ssp. *durum*	3.3	0.02	2.7	3.6	6.2	
	Landraces and old cultivars	3.2	0.05	2.7	3.6	8.2	
	Modern cultivars	3.3	0.02	3.1	3.6	4.6	
	ssp. *turanicum*	3.3	0.04	3.1	3.6	5.0	
	ssp*. polonicum*	3.4	0.04	3.2	3.7	5.1	
	ssp. *turgidum*	3.5	0.06	3.1	3.8	6.9	
	ssp. *carthlicum*	2.9	0.03	2.7	3.1	3.2	
	ssp. *dicoccum*	3.2	0.04	2.9	3.5	5.6	
	ssp. *dicoccoides*	2.8	0.07	2.5	3.2	8.4	
ASPECT	Whole collection	2.5	0.04	1.7	3.7	15.9	0.97
	ssp. *durum*	2.3	0.02	2.0	2.9	7.9	
	Landraces and old cultivars	2.4	0.05	2.0	2.9	10.6	
	Modern cultivars	2.3	0.02	2.1	2.6	5.7	
	ssp. *turanicum*	2.9	0.06	2.3	3.4	10.0	
	ssp*. polonicum*	2.7	0.05	2.3	3.0	7.9	
	ssp. *turgidum*	1.9	0.03	1.7	2.2	7.0	
	ssp. *carthlicum*	2.3	0.06	2.0	2.6	8.5	
	ssp. *dicoccum*	2.8	0.03	2.6	3.0	3.8	
	ssp. *dicoccoides*	3.2	0.09	2.6	3.7	9.5	

* Number of accessions of whole collection and of *T. turgidum* ssp. reported in round brackets of TKW.

The GL range in the third sentence of the second paragraph under the “3.1 Phenotypic variation for grain traits” section, “In the whole collection, a large variation was observed for TKW (26.1–81.3 g), AREA (12.8–29.6 mm^2^), and GL (12.8–29.6 mm) with average values of 48.9 g, 20.6 mm, and 8.1 mm, respectively (Figure 2)” has been corrected to “In the whole collection, a large variation was observed for TKW (26.1–81.3 g), AREA (12.8–29.6 mm^2^), and GL (5.6–10.7 mm) with average values of 48.9 g, 20.6 mm, and 8.1 mm, respectively (Figure 2).”

We apologize for these errors.

