# Supplementary material for: Genome‐wide association study identifies QTL and candidate genes for grain size and weight in a Triticum turgidum collection
Source: Plant Genome. 2025 Jan 27;18(1):e20562. doi: 10.1002/tpg2.20562 (PMC11771687; doi:10.1002/tpg2.20562)
Supplement: Supplementary file 1 [file TPG2-18-e20562-s001.pdf]

**Supplemental Figure S1**. Manhattan and quantile–quantile plots generated using a mixed linear model (MLM) for: **a**) thousand kernels weight (TKW); **b**) area (AREA); **c**) grain length (GL); **d**) grain width (GW); **e**) grain aspect (ASPECT) in a tetraploid wheat collection across three years.

The significance level (−log10($p$) ≥3.0) is represented by the red dashed horizontal line. The *X*-axis shows the position of SNPs along the 14 chromosomes, with various colours indicating distinct chromosomes. The *Y*-axis shows the −log10(P) observed in each analysis.

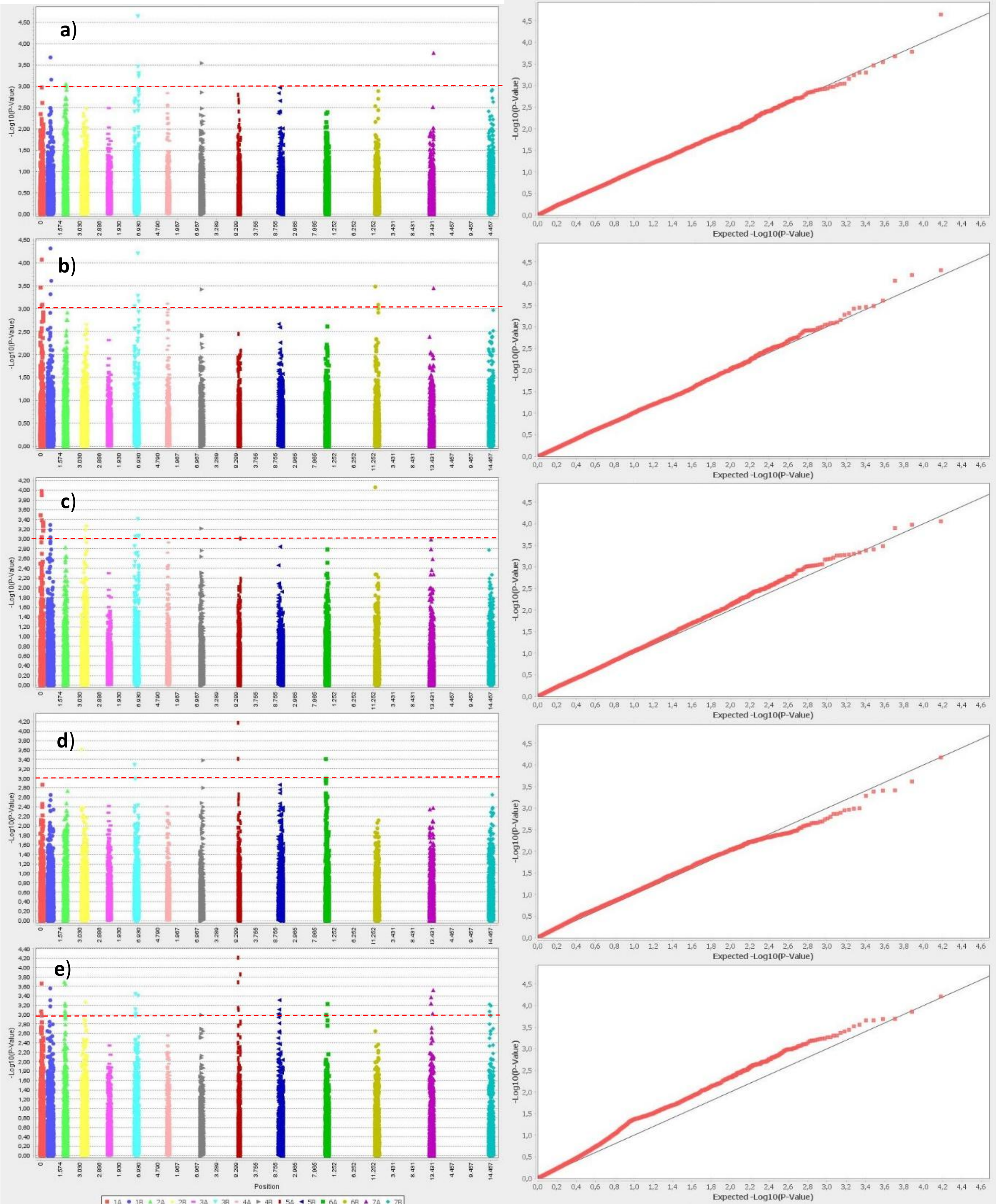

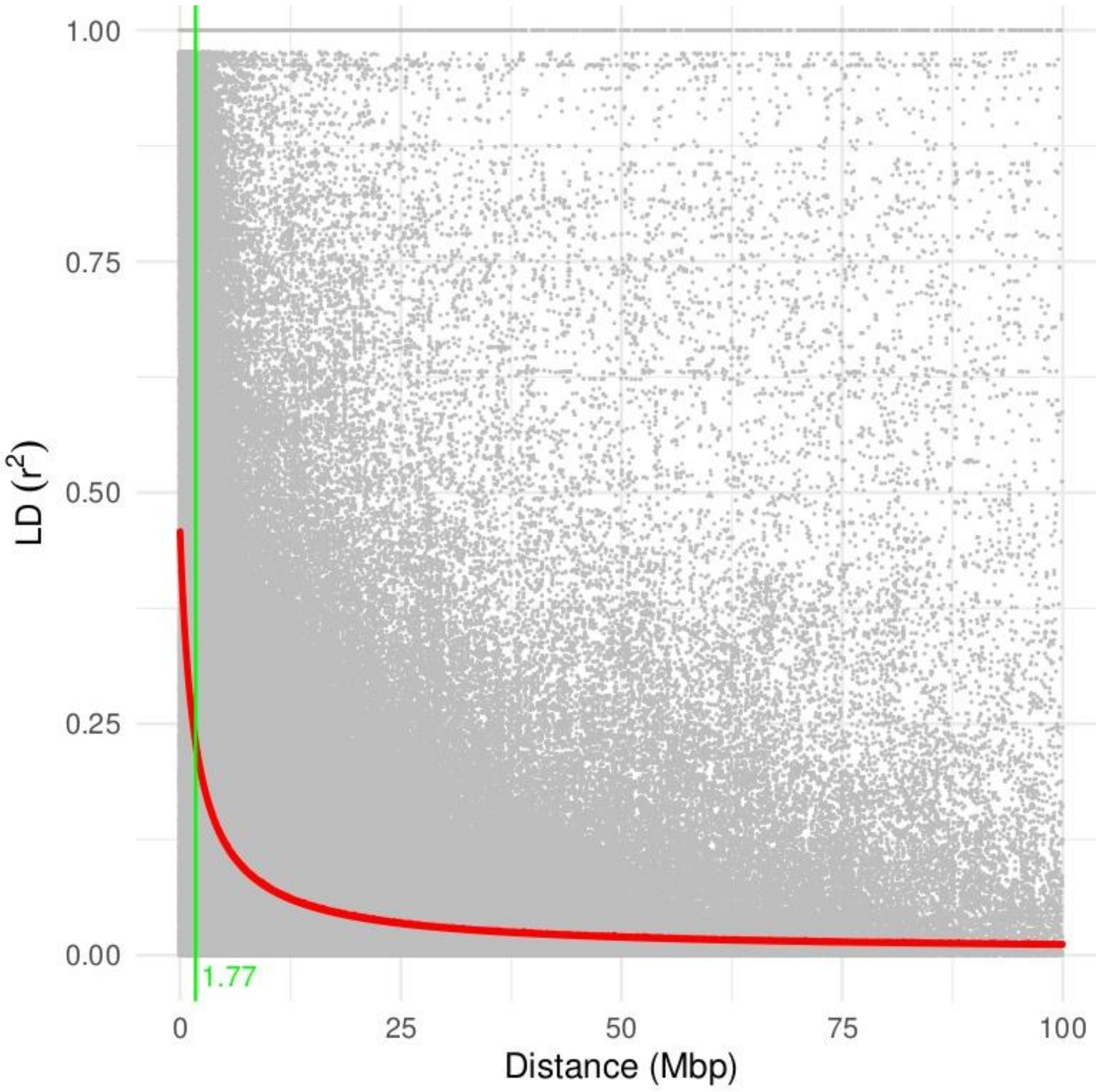


**Supplemental Figure S2**. Linkage disequilibrium (LD) decay in the tetraploid wheat collection. Pairwise LD ($r^2$) values plotted vs. corresponding pairwise physical distance (Mbp) of SNPs. The trend line of non-linear regression against physical distance is given by the red line. The point at which LD is reduced to 50% of its maximum value is indicated by the green vertical line.
