# Supplementary material for: Genome‐wide association study identifies QTL and candidate genes for grain size and weight in a Triticum turgidum collection
Source: Plant Genome. 2025 Jan 27;18(1):e20562. doi: 10.1002/tpg2.20562 (PMC11771687; doi:10.1002/tpg2.20562)
Supplement: Supplementary file 1 — Supplemental Figure S1. Manhattan and quantile–quantile plots generated using a mixed linear model (MLM) for (a) 1000‐kernel weight (TKW), (b) area (AREA), (c) grain length (GL), (d) grain width (GW), and (e) grain aspect (ASPECT) in a tetraploid wheat collection across 3 years. The significance level (−log10(p) ≥ 3.0) is represented by the red dashed horizontal line. The X‐axis shows the position of SNPs along the 14 chromosomes, with various colors indicating distinct chromosomes. The Y‐axis shows the −log10(p) observed in each analysis. Supplemental Figure S2. Linkage disequilibrium (LD) decay in the tetraploid wheat collection. Pairwise LD (r 2) values plotted versus corresponding pairwise physical distance (Mbp) of SNPs. The trend line of nonlinear regression against physical distance is given by the red line. The point at which LD is reduced to 50% of its maximum value is indicated by the green vertical line. [file TPG2-18-e20562-s001.pdf]

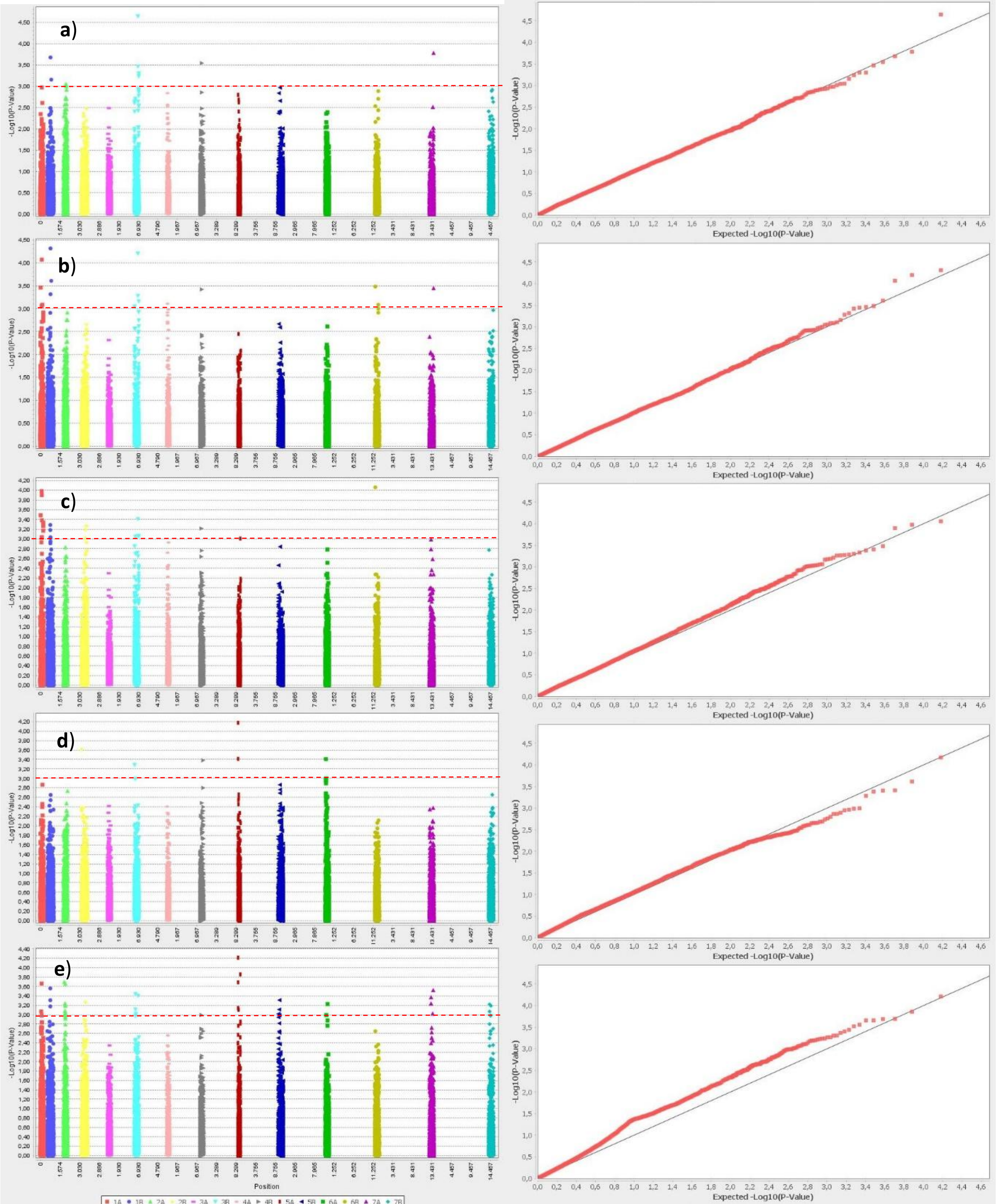

Supplemental Figure S1. Manhattan and quantile–quantile plots generated using a mixed linear model (MLM) for: **a**) thousand kernels weight (TKW); **b**) area (AREA); **c**) grain length (GL); **d**) grain width (GW); **e**) grain aspect (ASPECT) in a tetraploid wheat collection across three years.

The significance level (−log10(*p*) ≥3.0) is represented by the red dashed horizontal line. The *X*-axis shows the position of SNPs along the 14 chromosomes, with various colours indicating distinct chromosomes. The *Y*-axis shows the −log10(P) observed in each analysis.

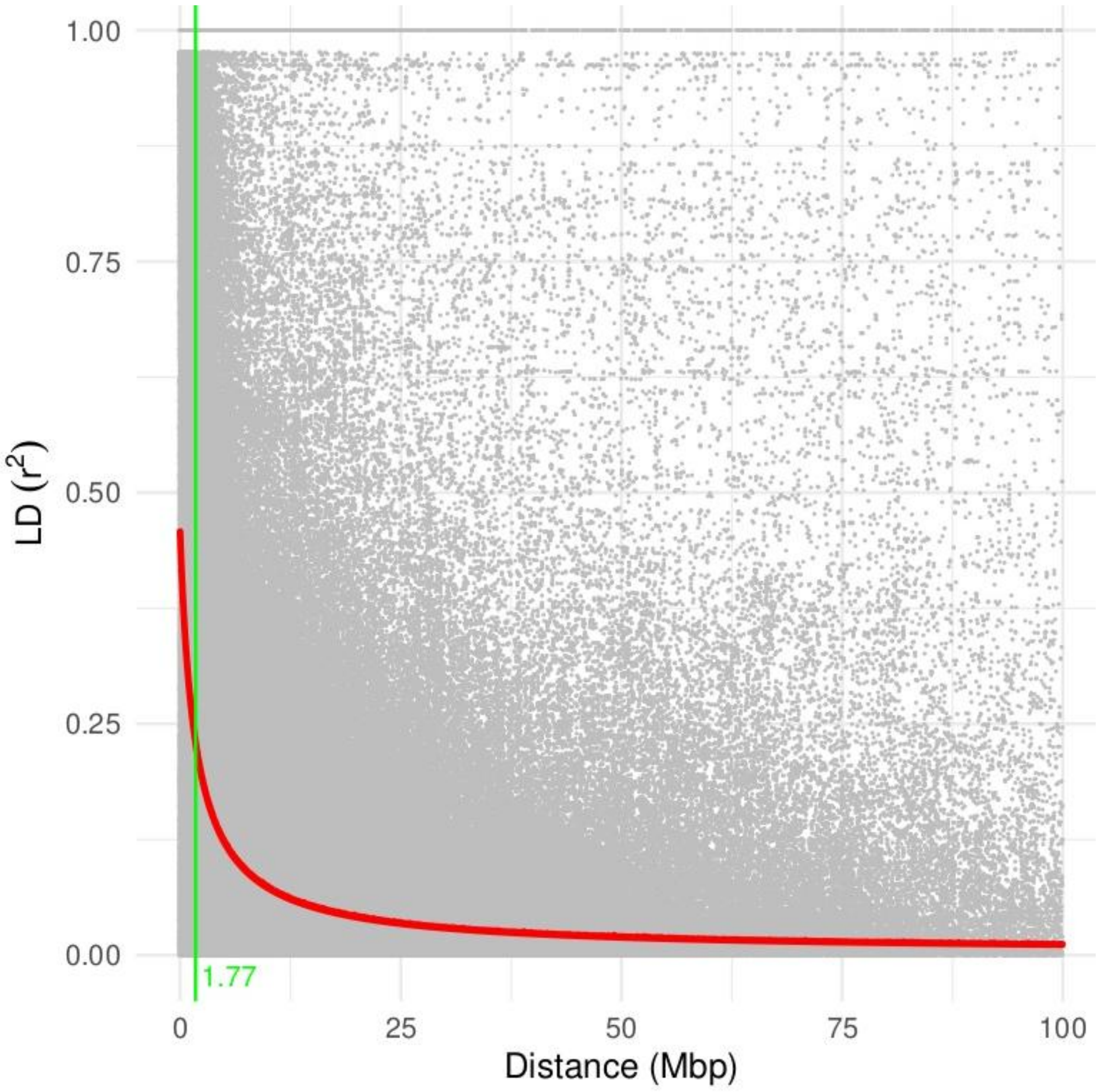

Supplemental Figure S2. Linkage disequilibrium (LD) decay in the tetraploid wheat collection. Pairwise LD (r^2) values plotted vs. corresponding pairwise physical distance (Mbp) of SNPs. The trend line of non-linear regression against physical distance is given by the red line. The point at which LD is reduced to 50% of its maximum value is indicated by the green vertical line.
